# The Bacterial MtrAB Two-Component System Regulates the Cell Wall Homeostasis Responding to Environmental Alkaline Stress

**DOI:** 10.1128/spectrum.02311-22

**Published:** 2022-09-08

**Authors:** Xiaoyu Qin, Kaiduan Zhang, Yuzhao Fan, Hui Fang, Yong Nie, Xiao-Lei Wu

**Affiliations:** a College of Engineering, Peking University, Beijing, China; b School of Resources and Environmental Engineering, Hefei University of Technology, Hefei, China; c Institute of Ocean Research, Peking University, Beijing, China; d Institute of Ecology, Peking University, Beijing, China; College of New Jersey

**Keywords:** *Dietzia* sp. DQ12-45-1b, two-component system, MtrAB, alkaline environment, MraZ, cell wall homeostasis, *Dietzia*

## Abstract

Throughout the course of evolution, bacteria have developed signal transduction tools such as two-component systems (TCSs) to meet their demands to thrive even under the most challenging environmental conditions. One TCS called MtrAB is commonly found in *Actinobacteria* and is implicated in cell wall metabolism, osmoprotection, cell proliferation, antigen secretion, and biosynthesis of secondary metabolites. However, precisely how the MtrAB TCS regulates the bacterial responses to external environments remains unclear. Here, we report that the MtrAB TCS regulates the cell envelope response of alkali-tolerant bacterium *Dietzia* sp. strain DQ12-45-1b to extreme alkaline stimuli. We found that under alkaline conditions, an *mtrAB* mutant exhibited both reduced growth and abnormal morphology compared to the wild-type strain. Electrophoretic mobility shift assay analysis showed that MtrA binds the promoter of the *mraZ* gene critical for cell wall homeostasis, suggesting that MtrA directly controls transcription of this regulator. In conclusion, our findings demonstrate that MtrAB TCS is involved in controlling the bacterial response to alkaline stimuli by regulating the expression of the cell wall homeostasis regulator MraZ in *Dietzia* sp. DQ12-45-1b, providing novel details critical for a mechanistic understanding of how cell wall homeostasis is controlled.

**IMPORTANCE** Microorganisms can be found in most extreme environments, and they have to adapt to a wide range of environmental stresses. The two-component systems (TCSs) found in bacteria detect environmental stimuli and regulate physiological pathways for survival. The MtrAB TCS conserved in *Corynebacterineae* is critical for maintaining the metabolism of the cell wall components that protects bacteria from diverse environmental stresses. However, how the MtrAB TCS regulates cell wall homeostasis and adaptation under stress conditions is unclear. Here, we report that the MtrAB TCS in *Dietzia* sp. DQ12-45-1b plays a critical role in alkaline resistance by modulating the cell wall homeostasis through the MtrAB-MraZ pathway. Thus, our work provides a novel regulatory pathway used by bacteria for adaptation and survival under extreme alkaline stresses.

## INTRODUCTION

Bacteria comprise approximately 15% of the entire biomass on Earth and can be found even in the most extreme environments where higher plants and animals fail to thrive (1). The cell envelope acts as a barrier to protect bacteria from external stresses, as well as from attacks. To maintain the integrity of the cell envelope in these extreme environments, bacteria utilize a wide variety of signal transduction regulatory systems, such as two-component systems (TCSs) (2). These TCSs are able to detect multiple environmental stimuli including pH, temperature, osmotic pressure, oxidative stress, and different ions (3–7). Prototypical TCSs typically consist of a histidine kinase sensor (HK) and a response regulator (RR). In general, the HK senses a stimulus, which leads to ATP hydrolysis and phosphorylation of a conserved histidine residue of the HK. Subsequently, the phosphorylated HK transfers the phosphoryl group to a conserved aspartic acid residue of the cognate RR, ultimately resulting in an activated form of the RR that in turn controls activation of genes further downstream (2, 8, 9).

The MtrAB comprises a highly conserved TCS that is specifically expressed in *Actinobacteria*. The MtrAB consists of MtrA, which functions as RR, and MtrB, which functions as HK (10, 11). The MtrAB TCS plays multiple roles in various cellular processes, including cell cycle control, cell wall homeostasis, and environmental stress resistance. For example, in Mycobacterium, the MtrAB TCS is essential and is involved in regulation of the cell cycle progression (i.e., replication and cell division), cell proliferation, cell wall homeostasis, and biofilm formation (12–14). Previous studies reported that the MtrA regulon comprises key players involved in cell division and cell wall metabolisms, such as *ftsI*, *dacB1*, *ripA*, *fbpBC*, and *rpfABC* (15–17). Two studies showed that MtrA interacts with the promoter of *dnaA* involved in DNA replication (18, 19). In contrast, the MtrAB TCS of Corynebacterium glutamicum is involved in osmoregulation and cell wall metabolism. The MtrAB TCS regulates cell wall metabolism via repression of *mepA* and *nlpC* and is involved in osmoregulation by directly activating *betP* and *proP*. (20, 21). Furthermore, in *Streptomyces* species, the MtrAB TCS plays an important role in the developmental life cycle (i.e., aerial mycelium formation and spore maturation). The phosphorylated MtrA regulates aerial mycelium formation by activating *chp*, *rdl*, and *ramCSAB* genes, as well as the *bldK* operon at the early phase. In addition, MtrA represses *bldD* to activate BldN, whose targets include *chp* and *rdl* genes, to regulate aerial mycelium formation. MtrA also affects *Streptomyces* differentiation, namely, by regulating *whiI* directly and *whiH* indirectly, both of which are essential for the activation of *whiE* (22). These observations strongly suggest that MtrAB TCS both directly and indirectly affects cell wall homeostasis, osmoprotection, cell cycle progression, and the developmental life cycle.

Peptidoglycan biosynthesis and metabolism represents an important adaptation to stress. For example, Exiguobacterium sibiricum increases the expression of peptidoglycan biosynthesis genes (*murADEI*) at −2.5°C, suggesting that the thickening of the cell wall may protect the cell against disruption by ice formation and/or osmotic pressure, on account of the subzero temperatures (23). In comparison, in Listeria monocytogenes, genes involved in peptidoglycan biosynthesis (*murABCE*) are downregulated, which is presumably related to the reduced cellular growth rate when experiencing cold stress (4°C) (24). In Mycobacteria, the PknB-CwlM-MurA regulatory pathway functions to swiftly repress peptidoglycan synthesis during nutrient limitation. This regulatory pathway possibly contributes to antibiotic tolerance during growth and under changing environmental conditions (25). Peptidoglycan modification is also involved in bacterial response to environmental pH perturbation. Increased expression of peptidoglycan biosynthesis has been reported for Escherichia coli under acid stress. This result possibly attempts to maintain cell wall structural integrity (26). In natural environments, bacteria usually encounter pH changes in the alkaline range, rather than acidic range. Several *Listeria monocytogenes* genes involved in cell division (i.e., *ftsE* and *iap*) are repressed, and the cell shape becomes filamentous or an elongated chain forms under alkaline stress. These changes in either cell division or peptidoglycan metabolism may represent an adaptive response resulting in resistance to alkaline growth conditions (27, 28). However, the effects on regulatory pathways responsible for changed peptidoglycan metabolism or cell division under alkaline pH stress remain to be elucidated.

The actinobacterial genus *Dietzia* is widely distributed in terrestrial and aquatic habitats and in clinical materials, even in the most extreme environments, such as deep seas and cold deserts (29–31). Most *Dietzia* species can grow under high-alkaline (pH 10 to 12), high-saline (7 to 16% [wt/vol] NaCl), and low-temperature (4°C) conditions. Moreover, *Dietzia* species have great potential for applications in a wide range of industries. They are thought to be good candidates for bioremediation in alkaline, salty, and frigid environments (32). In addition, *Dietzia* species were reported to be used as potential probiotics to inhibit Mycobacterium subsp. *paratuberculosis* growth in cultures *in vitro* (33). *Dietzia* sp. strain DQ12-45-1b is one of the most studied *Dietzia* strains; it was isolated from oil production water (34). It can degrade a wide range of aliphatic hydrocarbons and aromatic compounds and can grow under high-alkaline and high-saline conditions (34, 35). To understand its different adaptive mechanisms in alkaline environments, we set out to investigate the role of MtrAB TCS in cell wall homeostasis under the alkaline stress in *Dietzia* sp. DQ12-45-1b. We show that the MtrAB TCS controls the cellular shape and cell division in response to the alkaline stress, namely, by directly regulating the expression of the regulator MraZ, which contributes to cell wall metabolism and cell division.

## RESULTS

### MtrAB is essential for maintaining growth under alkaline conditions.

**FIG 1:**
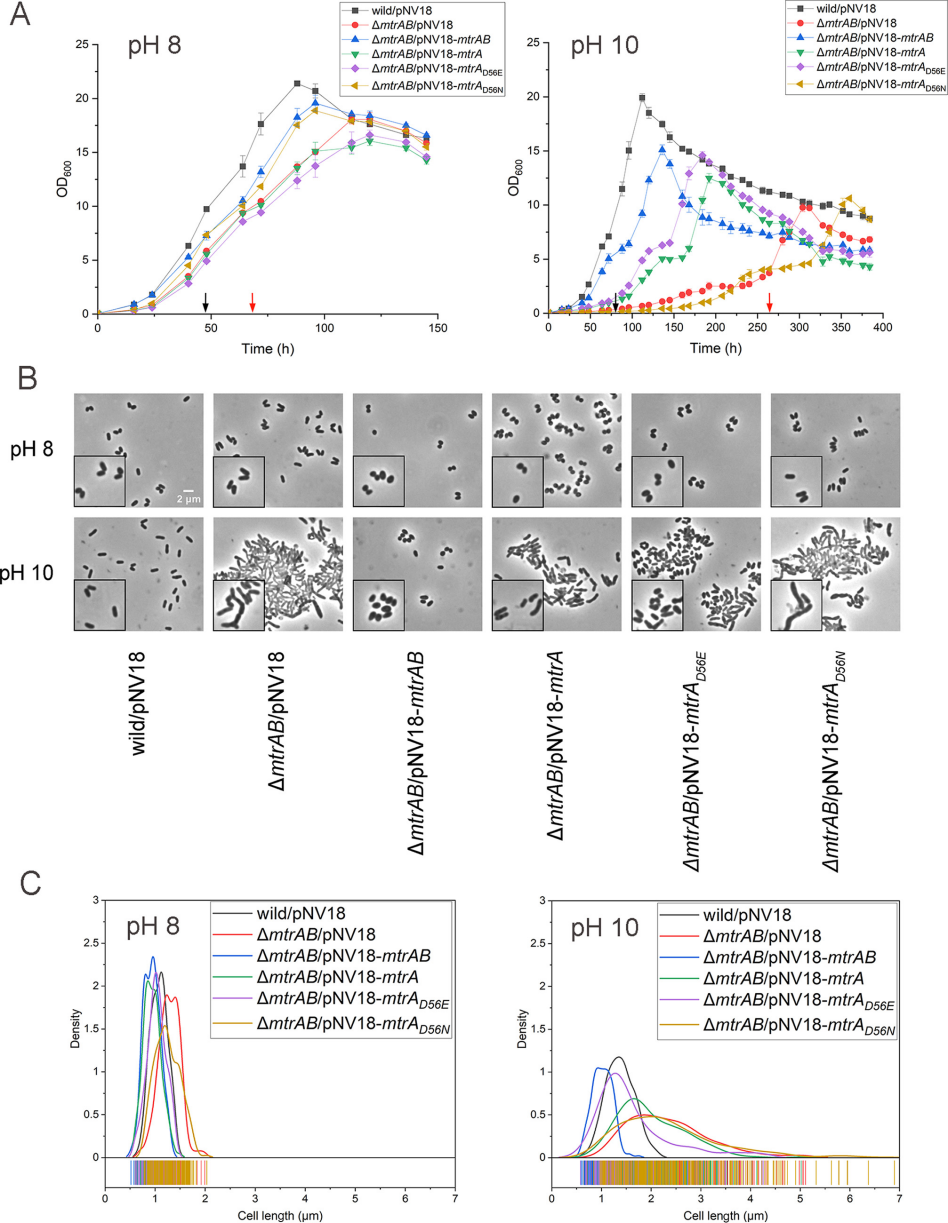
Characterization of Δ*mtrAB* mutants overexpressing wild-type, phosphorylation-defective, or phosphorylation-mimicking MtrA strains at different pHs. (A) Growth curves of the *Dietzia* sp. DQ12-45-1b wild-type strain carrying pNV18 or the Δ*mtrAB* mutant carrying pNV18 (empty plasmid), pNV18-*mtrAB*, pNV18-*mtrA*, pNV18-*mtrA_D56E_*, or pNV18-*mtrA_D56N_*, respectively, at pH 8 and pH 10. Sampling points for RNA-seq analyses of wild-type and Δ*mtrAB* mutant strains are indicated by black and red arrows, respectively. (B) Representative micrographs of the above-mentioned strains examined by bright-field microscopy. (C) Distribution curves of cell lengths at pH 8 and pH 10 of ~200 cells/sample from panel B were measured using Digimizer software.

*Dietzia* sp. DQ12-45-1b thrives under diverse environmental conditions, including extreme stress such as highly alkaline pH (34). *Dietzia* sp. DQ12-45-1b harbors 14 complete pairs of TCSs, as well as a gene encoding one orphan response regulator. We found that inactivation of the genes encoding MtrAB TCS homologs GJR88_01482/01483 significantly suppressed the growth rate at pH 10 (Fig. 1A). MtrA homolog GJR88_01482 in *Dietzia* sp. DQ12-45-1b (denoted as MtrA_Dz_) exhibits 81% amino acid sequence identity to MtrA of Mycobacterium tuberculosis, and GJR88_01483 (denoted as MtrB_Dz_) exhibits 56% identity to MtrB of M. tuberculosis. The complement of *mtrAB* genes recovered the growth of the Δ*mtrAB* mutant at pH 10 (Fig. 1A), suggesting that the TCS MtrAB plays a critical role in establishing resistance to alkaline conditions in *Dietzia* sp. DQ12-45-1b.

To investigate whether phosphorylation of MtrA is required for alkaline resistance in *Dietzia* sp. DQ12-45-1b, we compared the amino acid sequences of other potential MtrA and MtrB sequences from different genera. Our analysis showed that MtrA and MtrB from *Dietzia* sp. DQ12-45-1b contained highly conserved amino acid residues with other MtrAB proteins, including the potential phosphorylation sites at Asp56 of MtrA and His322 of MtrB (36, 37) (see Fig. S1 and S2 in the supplemental material). Next, we replaced the conserved Asp56 with either asparagine (MtrA_D56N_) to disrupt phosphorylation of MtrA or glutamate (MtrA_D56E_) to mimic phosphorylation (36). We found that the Δ*mtrAB* mutant strain overexpressing *mtrA_D56E_* partially restored the delayed growth at pH 10. In addition, overexpressing phosphorylation-defective *mtrA_D56N_* was similar to the Δ*mtrAB* mutant strain (Fig. 1A). These results suggested that phosphorylation of MtrA is required to establish resistance to alkaline stress in *Dietzia* sp. DQ12-45-1b. Importantly, overexpressing *mtrA* also restored the growth of the Δ*mtrAB* mutant strain at pH 10, suggesting that there is potential branched regulation of MtrA by other kinases under *in vivo* conditions.

### MtrAB is essential for maintaining regular cell morphology under alkaline conditions.

We found that under alkaline conditions, our Δ*mtrAB* mutant strain was irregularly shaped and elongated (Fig. 1B and C). The cell lengths of both wild-type strain and the Δ*mtrAB* mutant were similar at pH 8. However, at pH 10, the length of Δ*mtrAB* cells (2.79 ± 0.99 μm) was significantly (*P < *0.001) greater than that of wild-type cells (1.34 ± 0.23 μm) (Fig. 1B and C). We also noticed that the cells of Δ*mtrAB* mutant were irregularly shaped, with a small percentage exhibiting branched phenotypes (Fig. 1B; see also Fig. S3). At pH 10, the complement of *mtrAB* recovered cell length similar to that observed for the wild-type strain. Together, these results suggested that the MtrAB TCS is essential for maintaining normal cell morphology under alkaline stress conditions.

**FIG 2:**
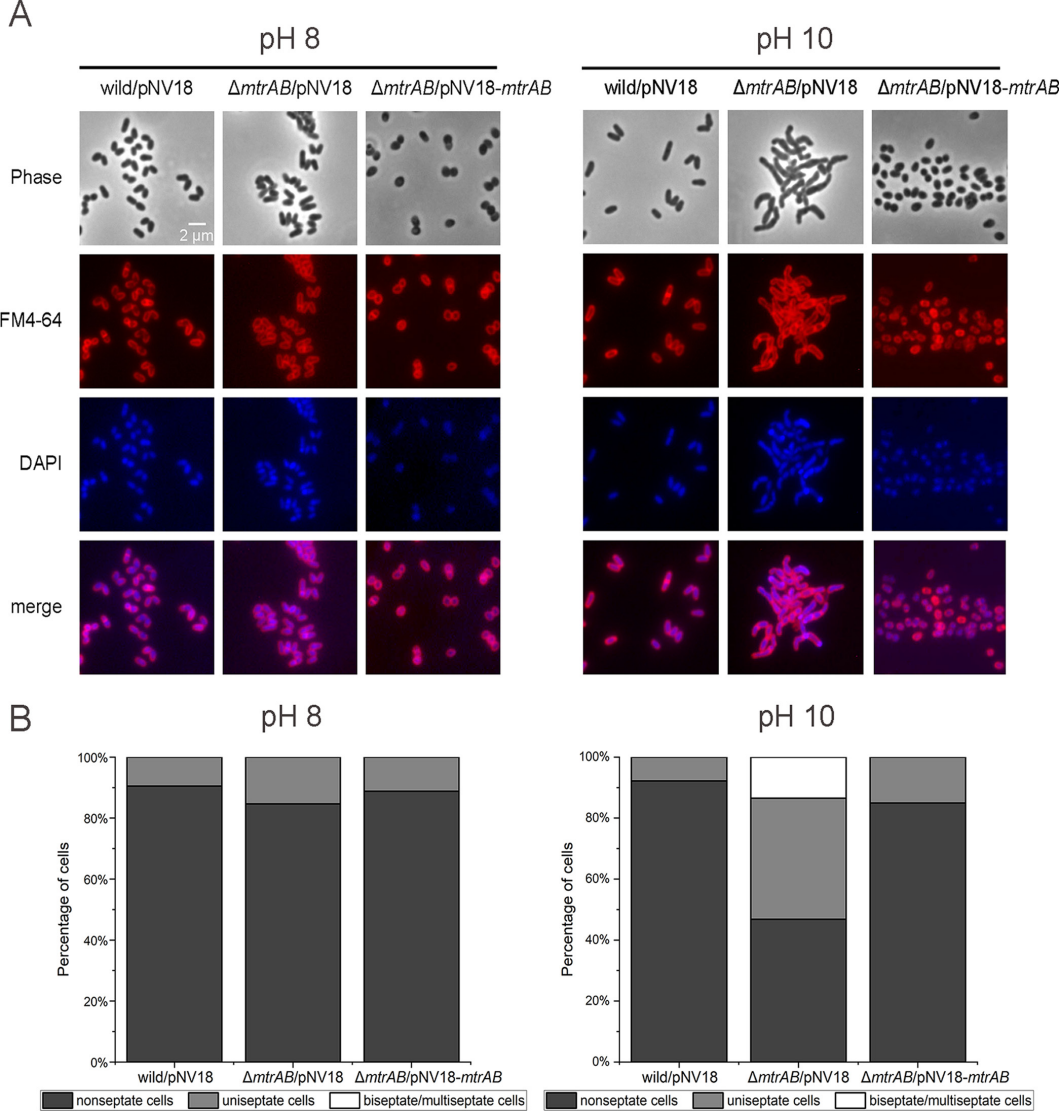
MtrAB TCS is required for septation in *Dietzia* sp. DQ12-45-1b at pH 10. (A) Fluorescence microscopy of septation in the *Dietzia* sp. DQ12-45-1b wild-type strain, the Δ*mtrAB* mutant, and complementary strain cells in the exponential growth phase at pH 8 and pH 10. The cell membrane and DNA were marked using FM4-64 (red) and DAPI (blue), respectively. (B) Stacked columns of the percentages of cells containing a different number of septa from A were analyzed at pH 8 and pH 10 (~200 cells/sample).

Dividing cells are known to possess one or more septa and DNA foci detectable by fluorescent labeling. Correspondingly, nonseptate cells (nondividing cells) are cells with a detectable gap between the membranes (38). To investigate whether elongation of Δ*mtrAB* cells was due to incomplete cell division, we used the fluorescent dyes FM4-64 and DAPI (4′,6′-diamidino-2-phenylindole) that detect the septa and DNA foci within dividing cells. Our results showed that nondividing cells dominated in all strains (i.e., the wild-type, Δ*mtrAB*, and complementary strains) at pH 8, which accounted for more than 80% of the total cells (Fig. 2). While this ratio significantly decreased in cells of Δ*mtrAB* at pH 10, it remained unchanged in cells of both wild-type and *mtrAB* complementary strains. Moreover, we detected multiple septa only in Δ*mtrAB* cells at pH 10, which account for ~13% of the total cells (Fig. 2B), indicating that the Δ*mtrAB* strain displayed cell division defects at pH 10. Furthermore, the complement of MtrA_D56N_ failed to recover the cell morphology of Δ*mtrAB* cells at pH 10. However, MtrA and MtrA_D56E_ partially recovered the cell shape of Δ*mtrAB* cells under alkaline conditions, with the phenotypes of the latter strain being closer to that of the wild-type strain (Fig. 1B and C). Together, these results indicated that the MtrAB TCS plays an important role in normal cell division under alkaline stress conditions.

### MtrAB regulates peptidoglycan metabolism in *Dietzia* sp. DQ12-45-1b.

**FIG 3:**
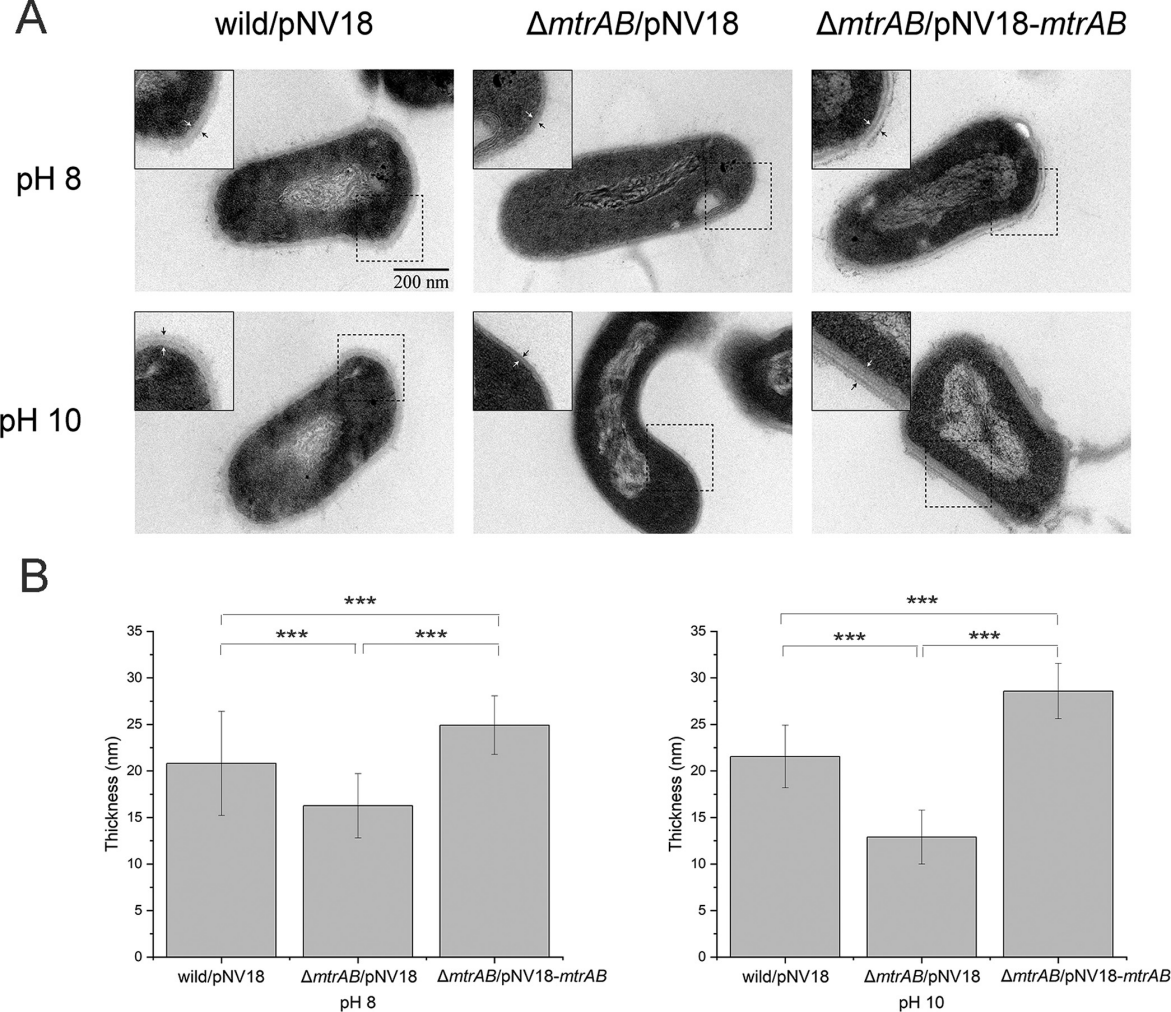
Ultrastructure of the cell envelope of *Dietzia* cells. (A) Representative thin-section transmission electron microscopy of the *Dietzia* sp. DQ12-45-1b wild-type strain, the Δ*mtrAB* mutant, and complementary strain cells under different pH conditions. The distance between two arrows represents the thickness of the cell envelope. Insets show small portions of cell cross sections for each image. (B) Histogram of cell envelope thickness measurements obtained by analyzing ~10 cells/sample in transmission electron microscopic cross sections from panel A. Every cross-section selected multiple regions for measurement using Digimizer (unpaired *t* test for panel B; ***, *P < *0.001).

Since the regulation of peptidoglycan synthesis plays an important role in bacterial growth and morphology (39), we speculated that MtrAB controls the growth and cell division under alkaline stress by regulating peptidoglycan metabolism. To probe the ultrastructure of the cell envelopes of strains under alkaline conditions, we used thin-section transmission electron microscopy. As shown in Fig. 3, deletion of *mtrAB* significantly (*P < *0.001) decreased the thickness of the cell envelope at both pH 8 and pH 10. Although high-pH conditions failed to affect the thickness of the cell envelope in the wild-type strain, they significantly reduced the thickness of the cell envelope in the Δ*mtrAB* mutant (Fig. 3). The change in the thickness of the cell envelope might result from a change in peptidoglycan metabolism, implying that MtrAB is involved in the regulation of peptidoglycan metabolism under alkaline stress conditions.

**FIG 4:**
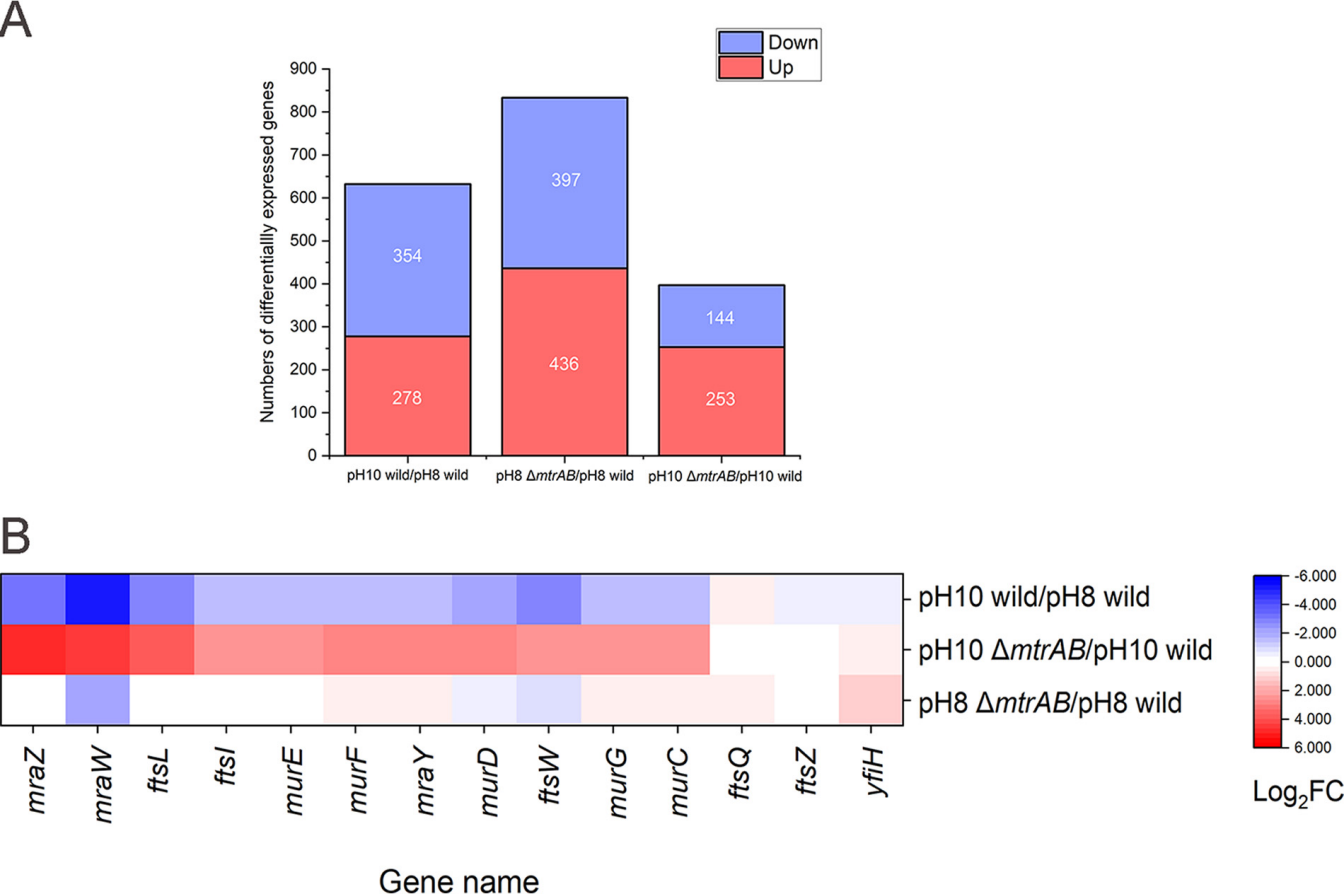
Transcriptomic analysis of the *dcw* cluster wild-type and Δ*mtrAB* mutant strains at pH 8 and pH 10. (A) Stacked columns of the number of DEGs (fold change ≥ 2, FDR ≤ 0.05). (B) Heatmap of the *dcw* cluster induced and repressed in wild-type and Δ*mtrAB* mutant strains under different pH conditions. Red represents upregulated genes, and blue represents downregulated genes.

To confirm the roles of *mtrAB* on the regulation of peptidoglycan metabolism under alkaline stress, we compared the transcriptional profiles of the wild-type and Δ*mtrAB* strains at different pH conditions. We found 278 upregulated genes and 354 downregulated genes of the wild-type strain at pH 10 compared to pH 8 conditions. In addition, we identified 144 genes with at least a 2-fold decrease and 253 genes with at least a 2-fold increase in the Δ*mtrAB* mutant compared to the wild-type strains under the alkaline stress (Fig. 4A). Based on the scatterplots, we confirmed that the parallel samples have a good linear correlation, suggesting that the data are reliable (see Fig. S4). To seek out the pathways altered by deletion of MtrAB under the alkaline stress, we analyzed transcriptomic data by Gene Ontology (GO) enrichment analysis (see Fig. S5). We found that a large number of enriched GO terms were related to cell division, cell wall biosynthesis, and metabolic process. Upon further analysis, we observed significantly downregulated expression of the genes involved in peptidoglycan biosynthesis (*murCDEFGJ*, *mraY*, and *ftsWI*), hydrolysis (*ripA*, *cwlO*, *mepA*), cell division (*ftsEXKL*, *sepF*, and *sepIVA*), and related regulators (*mraZ*, *envC*, and *whmD*), especially the division cell wall (*dcw*) gene cluster in the wild-type strain at pH 10 compared to pH 8 (Fig. 4B; see also Table S1). These results implied that the regulation of peptidoglycan synthesis, hydrolysis, and cell division is important for alkaline resistance in the wild-type strain. The Δ*mtrAB* mutant exhibited significantly higher expression of the above-mentioned genes compared to wild-type strain at pH 10 (Fig. 4B; see also Table S1). The results suggested that MtrAB represses the genes involved in peptidoglycan homeostasis and cell division, especially the *dcw* gene cluster under alkaline growth conditions. Next, we overexpressed these genes involved in peptidoglycan homeostasis, cell division, and related regulators in the *Dietzia* sp. DQ12-45-1b wild-type strain. However, these overexpressed strains failed to cause abnormal cell morphology except the *mraZ*-overexpressing strain (see Fig. S6 and S7), indicating that at pH 10, a number of genes participating in peptidoglycan synthesis, hydrolysis, and cell division simultaneously act on the phenotypes of Δ*mtrAB* mutant. Together, these results suggested that MtrA represses specific genes involved in peptidoglycan homeostasis and cell division at pH 10, presumably to ensure that *Dietzia* sp. DQ12-45-1b maintains normal cell morphology.

### MtrAB controls cell wall homeostasis at pH 10 by directly repressing *mraZ* gene expression.

**FIG 5:**
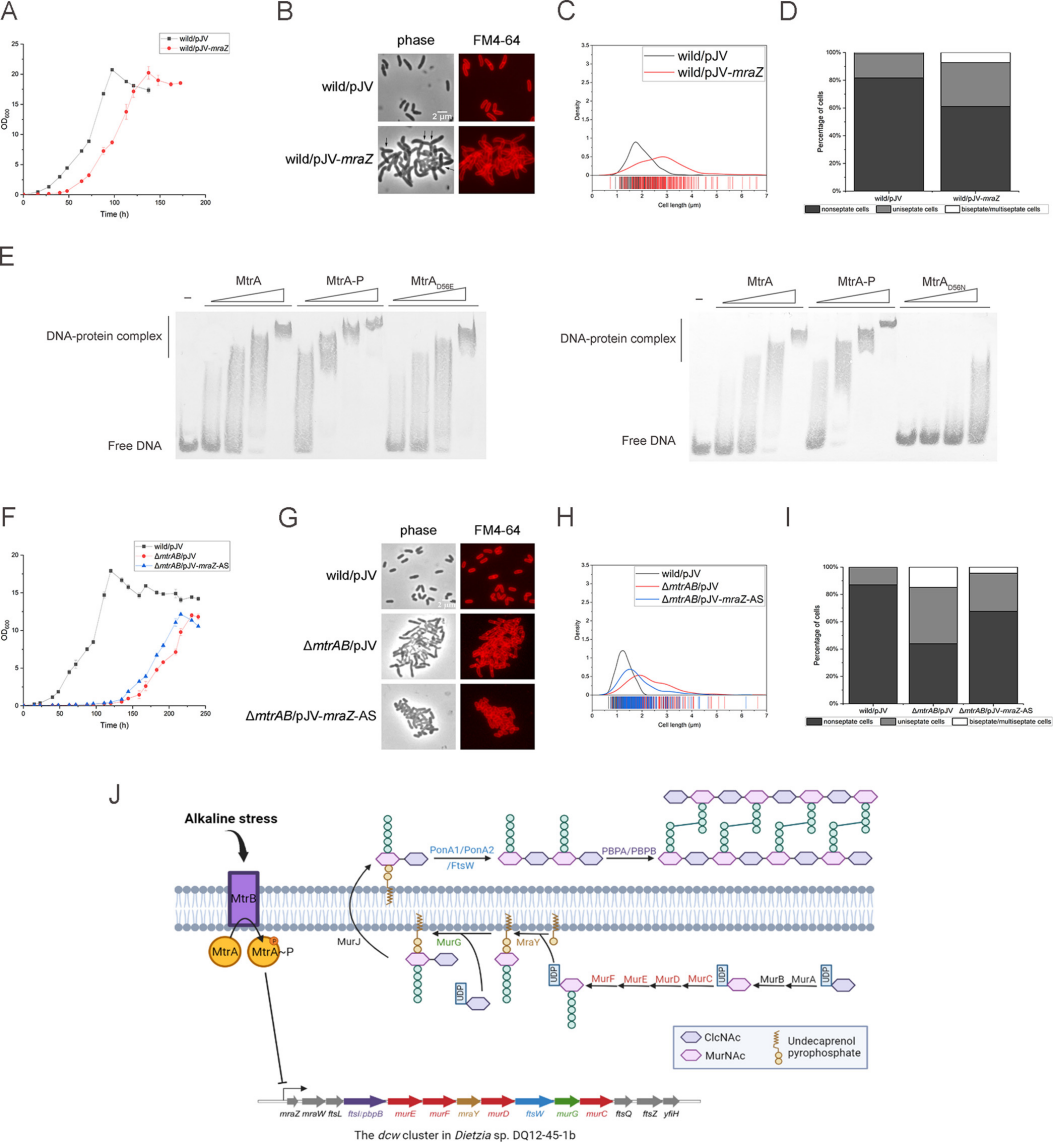
Characterization of wild-type strains overexpressing *mraZ*, Δ*mtrAB* strains silencing *mraZ* at pH 10 stress, and binding of MtrA to the *mraZ* promoter analyzed by EMSA. (A and F) Growth curves of the *Dietzia* sp. DQ12-45-1b wild-type strain containing the *mraZ* overexpression plasmid and the Δ*mtrAB* strain containing antisense mRNA of *mraZ* plasmid from the acetamide-inducible promoter at pH 10. (B and G) Fluorescence microscopy of septation in wild type/pJV-*mraZ* (wild/pJV-*mraZ*) and Δ*mtrAB*/pJV-*mraZ*-AS under pH 10 stress. The branched phenotype is marked with arrows. (C, D, H, and I) Distribution curves and stacked columns of the percentage of cells containing a different number of septa from panels B and G were analyzed (~200 cells/sample). (E) EMSA analysis of MtrA, MtrA-P, MtrA_D56E_, and MtrA_D56N_ binding to the *mraZ* promoter. MtrA, MtrA_D56E_, MtrA_D56N_, and MtrA~P at 0.5, 1, 2.5, and 5 μM were incubated for 30 min with 10 pM DIG-labeled P_mraz_. (J) Proposed mechanism’s diagram illustrating the relationship between MtrAB TCS and the *dcw* cluster in *Dietzia* sp. DQ12-45-1b.

Since *mraZ* is the head gene of the *dcw* cluster that potentially regulates downstream genes (Fig. 4B) (40), we wondered whether MtrAB regulates the expression of the *mraZ* gene in response to alkaline stress in *Dietzia* sp. DQ12-45-1b. We first confirmed the function of MraZ by overexpressing this gene in the *Dietzia* sp. DQ12-45-1b wild-type strain. We found that at pH 10, the strain overexpressing the *mraZ* gene exhibited a significantly delayed growth and an elongated and partially branched cell shape (Fig. 5A to C). We also noticed that overexpression of the *mraZ* gene resulted in the formation of multiseptate cells. These cells comprised approximately 7% of the total population during the early exponential phase at pH 10 (Fig. 5B and D). These results clearly demonstrated that overexpression of MraZ results in morphological deformation together with delayed growth. Since the expression of *mraZ* was induced in the Δ*mtrAB* mutant at pH 10, these results suggested that the function of the MtrAB TCS is to repress the expression of MraZ to maintain cell wall homeostasis under alkaline stress.

To investigate whether MtrA protein regulates *mraZ* directly, we prepared MtrA protein, phosphorylation-defective MtrA_D56N_, and phosphorylation-mimic MtrA_D56E_, phosphorylated the MtrA with EnvZ histidine kinase *in vitro*, and carried out an electrophoretic mobility shift assay (EMSA) with a digoxigenin (DIG)-labeled 100-bp DNA fragment upstream of the *mraZ* gene. We found that when the protein concentrations were up to 5 μM for MtrA and MtrA_D56E_ and 2.5 μM for phosphorylated MtrA, a slower shift band was observed, demonstrating that both the MtrA and the phosphorylated MtrA directly bind to the promoter region of the *mraZ* gene (Fig. 5E). On the other hand, we found that MtrA_D56N_ fails to bind to the promoter of *mraZ*. Previous studies reported that the replacement of the phosphorylation site of the response regulator with asparagine does not significantly affect protein conformation (36, 41), so the functional change of D56N mutant may result from the disruption of the phosphorylation site. Moreover, MtrA and MtrA-P cannot bind to the negative-control DNA (see Fig. S8). Compared to phosphorylation-defective MtrA_D56N_, MtrA can bind to the probe. One possible reason is that MtrA is slightly phosphorylated by other kinase sensors in Escherichia coli before being purified because of HK-RR branched regulation (42). Together, these results strongly suggested that phosphorylation is required for the MtrA-DNA binding activity, at least under our *in vitro* conditions.

We next disrupted the expression of the *mraZ* gene in the Δ*mtrAB* mutant to assess whether repression of MraZ is necessary for the regulation of MtrAB in responding to alkaline stress. We failed to delete the *mraZ* gene in the Δ*mtrAB* mutant. We speculated that the *mraZ* gene is essential in the Δ*mtrAB* mutant and then silenced the *mraZ* gene in the Δ*mtrAB* mutant under the control of the acetamide-inducible promoter through antisense RNA technology (43, 44). We found that under pH 10 conditions, the cell morphology of Δ*mtrAB* containing antisense mRNA of *mraZ* was partially recovered when grown with 0.5% acetamide, but the growth was similar to that observed with the Δ*mtrAB* mutant strain (Fig. 5F to I). This result suggested that MraZ participates in both peptidoglycan biosynthesis and cell division of MtrAB under alkaline stress. Together, since MtrAB directly regulates the *mraZ* gene, we believe that MtrAB controls cell wall homeostasis under alkaline stress by directly repressing the *mraZ* gene (Fig. 5J).

## DISCUSSION

In this study, we demonstrated that the MtrAB TCS regulates the cell wall homeostasis in *Dietzia* sp. DQ12-45-1b responding to environmental alkaline stress, namely, in a MraZ-dependent manner. This is the first report showing that the MtrAB TCS of *Dietzia* bacteria plays a critical role in peptidoglycan biosynthesis and cell division under environmental alkaline stress conditions.

The MtrAB TCS maintains the cell wall homeostasis which may be related to the universal responses to diverse environmental stresses. For example, in Mycobacterium tuberculosis, MtrB is required to withstand acid stress (pH 5.5) (14). In Corynebacterium glutamicum MtrAB is involved in osmoregulation, as well as cell wall metabolism (20). Here, we also demonstrated that MtrAB is essential for maintaining growth under alkaline condition in *Dietzia* sp. DQ12-45-1b. In addition, based on the transcriptomic data, we found that the expression levels of *mtrA* and *mtrB* increased ~1.5-fold (<2-fold) in pH 10 compared to pH 8 in the wild-type strain (see Table S1). Moreover, in *Dietzia* sp. DQ12-45-1b, deletion of *mtrAB* also resulted in growth defect, increased cell length, and branched cell shape in response to various cell envelope stresses, such as oxidative, sodium dodecyl sulfate (SDS), hyperosmotic, and cell-envelope-targeting antibiotic stresses (data not shown). These findings suggest that MtrAB may play a critical role in response to the diverse environmental stresses. Interestingly, the MtrAB deficiency also resulted in abnormal cell morphology in the above-mentioned bacteria. It has been reported previously that Δ*mtrA* and Δ*mtrB* mutants of Mycobacterium smegmatis both exhibit filamentous with branches and buds (15, 45). In addition, a Δ*mtrAB* mutant form of Corynebacterium glutamicum exhibited increased cell length and irregular septum formation (20). In addition, the absence of *mtrB* in Mycobacterium tuberculosis resulted in a chain-like phenotype and increased bulbous regions (13, 37). In summary, deletion of MtrAB TCS results in elongated cells with irregular septa in *Corynebacterineae*, indicating that the universal pathways that MtrAB TCS regulates may play a critical role in cell morphology. These findings are also important for the future research of the signals recognized by MtrB. MtrAB TCS is involved in response to diverse cell envelope stresses and regulates cell wall homeostasis, which suggests that cell wall fragments are sensed by MtrB and are likely to be universally present in *Corynebacterineae*.

Based on our transcriptomic analysis, we found that at pH 10 condition, the expression levels of genes involved in peptidoglycan biosynthesis (*murCDEFGJ*, *mraY*, and *ftsWI*), hydrolysis (*ripA*, *cwlO*, and *mepA*), cell division (*ftsEXKL*, *sepF*, and *sepIVA*), and related regulators (*mraZ*, *envC*, and *whmD*) significantly changed in Δ*mtrAB* mutant compared to the wild-type strain, a process that was accompanied by the appearance of irregular cell morphology, as well as delayed growth, in our Δ*mtrAB* mutant. It is well known that cooperation between peptidoglycan synthetases and hydrolases is critical for cell elongation and division (46, 47). In addition, the enzymes involved in peptidoglycan synthesis and hydrolysis are coregulated to ensure the balance between two processes (25, 48, 49). It also has been reported that imbalance between peptidoglycan synthesis and hydrolysis results in cell lysis (50). Therefore, abnormal transcriptional changes of genes involved in peptidoglycan synthesis and hydrolysis might lead to *Dietzia* sp. DQ12-45-1b Δ*mtrAB* cell lysis, thereby reducing the rate of growth. Moreover, the transcriptomic analysis showed that the expression levels of previously known sodium/proton antiporter genes such as *mrpACDEFG* related to alkaline resistance did not significantly change in the Δ*mtrAB* mutant (see Table S1), which suggested that MtrAB regulated a novel pathway for alkaline resistance (35). Although the mechanisms need to be further determined, the abnormal cell morphology of *mtrAB*-deficient strains in *Corynebacterineae* suggested that the cell wall homeostasis maintained by MtrAB may be related to the responses to alkaline stress, even to other diverse cell envelope stress conditions.

The MtrA-MraZ regulatory pathway may be universal but not exclusive to *Corynebacterineae*. Based on our findings, we conclude that MtrAB TCS in our *Dietzia* sp. DQ12-45-1b strain is directly involved in peptidoglycan biosynthesis, as well as cell division, that depends on the regulator MraZ during responses to alkaline stress. Since *mraZ* has been reported to belong to putative MtrA targets using a global CHIP-seq method in Mycobacterium tuberculosis (15), the MtrA-MraZ regulatory pathway may represent one universal pathway used by *Corynebacterineae* to achieve normal cell morphology. However, our results also suggest that the MtrA-MraZ regulatory pathway is not an exclusive pathway in response to alkaline stress. We showed that the phenotypes of overexpression of *mraZ* were similar to the Δ*mtrAB* mutant, but silencing of *mraZ* in the Δ*mtrAB* mutant was partially offset in response to alkaline stress. Together, these results suggest that additional proteins in addition to MraZ levels are critical for the phenotype of our Δ*mtrAB* mutant strain under alkaline conditions. Transcriptomic analysis showed that other genes belonging to the MtrA regulon, such as *mepA* and *whmD*, were also upregulated in Δ*mtrAB* mutant strain under alkaline conditions (15, 20). Previous studies reported that overexpression of the hydrolase gene *mepA* in Escherichia coli resulted in an elongated cell morphology, presumably because of interference with the localization or activity of certain PG synthetases (20, 51). Overexpression of the regulator gene *whmD* in the Mycobacterium smegmatis wild-type strain resulted in mislocated septa, presumably because WhmD levels are important for the efficiency or correct location of septum formation, perhaps by affecting FtsZ localization or assembly (52, 53). However, in contrast to previous reports, overexpression of either MepA or WhmD in *Dietzia* sp. DQ12-45-1b failed to change cell morphology (see Fig. S7). These results suggest that MtrAB regulates redundant pathways to maintain the cell wall homeostasis and normal cell morphology to respond to the environmental stresses.

In conclusion, the relationship of MtrAB TCS and MraZ in this study should greatly assist future investigations into the transcriptional regulators of MraZ. We established here the direct relationship between MtrAB TCS and MraZ involved in cell wall synthesis and cell division. The *dcw* cluster, including the *mraZ* gene, is well conserved in most bacteria and thus is important for investigating the regulatory pathways of the *mraZ* gene in more detail.

## MATERIALS AND METHODS

### Bacterial strains and growth conditions.

The strains and plasmids used are listed in Table S2 in the supplemental material.

All *Dietzia* strains were cultured in GPY medium (glucose [10 g/L], tryptone [10 g/L], and yeast extract [5 g/L]) with the appropriate antibiotics (kanamycin [50 μg/mL] and streptomycin [30 μg/mL]) at 30°C and shaken at 150 rpm. For pH stress experiments, GPY medium was added with a mixture of 0.2 M Na_2_HPO_4_·12H_2_O and 0.2 M NaH_2_PO_4_·2H_2_O to pH 8 and with a mixture of 0.2 M Na_2_CO_3_ and 0.2 M NaHCO_3_ to pH 10. To eliminate the effects of different sodium concentrations, we added 0.05 M NaCl into pH 10 GPY medium. All Escherichia coli strains were cultured in Luria-Bertani (LB) medium (NaCl [10 g/L], tryptone [10 g/L], and yeast extract [5 g/L]) with the appropriate antibiotics (kanamycin [50 μg/mL]) at 37°C with shaking at 150 rpm. Since the differences in cell length had little effect on the optical density measurement, we monitored the growth by measuring optical density instead of CFU. We measured the optical density at 600 nm (OD_600_) by using a UV spectrophotometer (Shimadzu, Japan). All growth curves experiments were performed in three biological replicates. Moreover, we added 0.5% acetamide to cultures of the strains harboring pJV-related plasmids to exclude the potential effects of acetamide.

### Construction of the Δ*mtrAB* mutant and complementary strains.

The preparation method of electrocompetent cells of *Dietzia* sp. DQ12-45-1b was described previously (35). Briefly, *Dietzia* strains were cultured at OD_600_ of 0.05 in the presence of the appropriate antibiotics and 0.5% glycine overnight at 30°C with 150-rpm shaking. We added 0.2% Tween 80, penicillin (0.5 μg/mL), and isoniazid (0.8 mg/mL) to the cultures when they had grown to an OD_600_ of 0.4 to 0.6. In addition to these substances, 0.2% acetamide was added to the culture of the *Dietzia* sp. DQ12-45-1b wild type/pJV53 (wild/pJV53) strain. After 4 h, the cells were harvested and washed twice with 10% glycerin by centrifugation at 1,500 × *g* for 10 min at 4°C. The electrocompetent cells were resuspended in 10% glycerin and stored at −80°C for further experiments.

The Δ*mtrAB* mutant strain was obtained by the double homologous recombination method (54). In brief, the upstream and downstream homologous fragments of *mtrAB* (~500 bp) were PCR amplified from the genomic DNA of *Dietzia* sp. DQ12-45-1b using the primer pairs mtrABLF/mtrABLR and mtrABRF/mtrABRR (see Table S3). Next, two homologous fragments were fused to a streptomycin cassette by PCR using the primers fusionF and fusionR (see Table S3). The fused DNA fragment containing the upstream and downstream homologous regions of *mtrAB* flanking the streptomycin cassette was then transformed into *Dietzia* sp. DQ12-45-1b wild/pJV53 competent cells by electroporation (2,000 V/mm, 12 ms) (55, 56). Using this method, *mtrAB* was successfully replaced by the streptomycin cassette to obtain the Δ*mtrAB* mutant strain.

The target genes *mtrA* and *mtrAB* were amplified from genomic DNA of *Dietzia* sp. DQ12-45-1b using the primer pairs mtrAF/mtrAR and mtrAF/mtrABR with HindIII restriction sites on both sides. Next, the DNA fragment of the vector pNV18 was amplified with the HindIII restriction sites using the primers pNVF and pNVR from the pNV18-DsRed plasmid to delete the gene encoding DsRed (see Table S3). The HindIII-digested DNA fragments of *mtrA* and *mtrAB* were then ligated into the HindIII-digested fragment from pNV18-DsRed. The recombinant plasmids pNV18-*mtrA* and pNV18-*mtrAB* were transformed into Δ*mtrAB* mutant competent cells by electroporation to produce the complementary strains.

### Construction of acetamide-inducible *mraZ* overexpression vector.

The complete *mraZ* gene fragment was cloned from genomic DNA of *Dietzia* sp. DQ12-45-1b by PCR using the primers mraZF and mraZR. Also, the DNA fragment of the vector pJV was amplified using the primers pJVF and pJVR from the pJV53 plasmid (see Table S3). Next, the *mraZ* PCR fragment was ligated into pJV, which contains an acetamide-inducible promoter, using the homologous recombination method with a Hieff Clone Plus One Step cloning kit (Yeasen). *Dietzia* sp. DQ12-45-1b was electroporated with the recombinant overexpression plasmid pJV-*mraZ*.

### Construction of *mraZ* antisense expression vector.

Portion of the *mraZ* gene was amplified from genomic DNA of *Dietzia* sp. DQ12-45-1b by PCR using the primers mraZASF and mraZASR. Next, the plasmid vector pJV53 was linearized by PCR using the primers pJVF and pJVR (see Table S3). The purified *mraZ* PCR fragment was ligated into pJV in an antisense orientation using the same homologous recombination method by Hieff Clone Plus One Step cloning kit (Yeasen). The recombinant plasmid pJV-*mraZ*-AS was obtained, whose expression can be induced by acetamide. The pJV-*mraZ*-AS was electroporated into *Dietzia* sp. DQ12-45-1b Δ*mtrAB* mutant competent cells.

### Construction of overexpression vectors carrying genes involved in peptidoglycan homeostasis and cell division.

All genes involved in peptidoglycan homeostasis and cell division were cloned from genomic DNA of *Dietzia* sp. DQ12-45-1b by PCR using the primers shown in Table S3. The plasmid vector pNV18 was linearized by PCR using the primers pNVF2 and pNVR2 (see Table S3). Next, cell wall-related genes were ligated into pNV18, which contains a p45 promoter, using the Hieff Clone Plus One Step cloning kit (Yeasen) homologous recombination method. All recombinant overexpression vectors were electroporated into *Dietzia* sp. DQ12-45-1b wild-type competent cells.

### Construction of *mtrA* phosphorylation-defective and phosphorylation-mimicking vectors.

The methods which cause *mtrA* to lose and mimic phosphorylation activities by point mutant were already verified (36). The primers mtrAD56NF/mtrAD56NR and pNVD56NF/pNVD56NR were used to amplify by PCR from pNV18-*mtrA* plasmid to replace the aspartic acid at codon 56 with asparagine to create plasmid pNV18-*mtrA_D56N_*. Next, the primer pairs mtrAD56EF/mtrAD56NR and pNVD56NF/pNVD56ER were used to amplify from pNV18-*mtrA* to replace the aspartic acid at codon 56 with glutamate to create plasmid pNV18-*mtrA_D56E_* (see Table S3). To construct the vectors pNV18-*mtrA_D56N_* and pNV18-*mtrA_D56E_*, homologous recombination was performed using a Hieff Clone Plus One Step cloning kit. Two recombinant plasmids were electroporated into *Dietzia* sp. DQ12-45-1b Δ*mtrAB* mutant competent cells.

### Construction of EnvZ_del_, MtrA, MtrA_D56E_, and MtrA_D56N_ expression vectors.

To purified EnvZ_del_, the region of *envZ* without transmembrane region (*envZ_del_*) was PCR amplified from E. coli K-12 substrain MG1655 with the primers envZF and envZR. The plasmid vector pET-28a was linearized by PCR using the primers pETF and pETR (see Table S3). The purified *envZ_del_* PCR fragment was ligated into pET-28a using the same homologous recombination method by the Hieff Clone Plus One Step cloning kit. For purification of the MtrA protein, the complete *mtrA* gene was cloned from *Dietzia* sp. DQ12-45-1b using the primers mtrAF and mtrAR incorporating NdeI and HindIII restriction sites, respectively. Next, the *mtrA_D56E_* and *mtrA_D56N_* fragments were cloned from pNV18-*mtrA_D56E_* and pNV18-*mtrA_D56N_* plasmids using the same primers, mtrAF and mtrAR. Simultaneously, the vector pET-28a was linearized using the primers pETF2 and pETR2 (see Table S3). The amplified *mtrA*, *mtrA_D56E_*, and *mtrA_D56N_* DNA fragments were ligated into the NdeI- and HindIII-digested pET-28a fragment. Then, the recombinant plasmids pET-28a-*envZ_del_*, pET-28a-*mtrA*, pET-28a-*mtrA_D56E_*, and pET-28a-*mtrA_D56N_* were transformed into the E. coli expression strain BL21(DE3) (TransGen).

### Transmission electron microscopy.

Cells that grew to the midlog phase were collected by centrifugation at 5,000 rpm for 5 min at 4°C after being washed three times with phosphate-buffered saline (PBS; pH 7.4) and then fixed with fresh 2.5% glutaraldehyde overnight at 4°C. The cells were then rinsed three times and concentrated with ddH_2_O. The cells were placed on a carbon-filmed, 300-mesh copper grid (Electron Microscopy China) for 2 min. Next, the grids were negatively stained using 2% (wt/vol) uranyl acetate in water for 1 min. The excess sample was removed using Whatman filter paper. Grids were examined using a transmission electron microscope (Hitachi HT7700, Japan) operated at 80 kV.

### Bright-field and fluorescence microscopy.

For staining procedures, cells were washed three times with PBS buffer (pH 7.4) and collected by centrifugation at 5,000 rpm for 5 min at 4°C. Membrane dye FM4-64 (Thermo Fisher) and DNA dye DAPI (Beyotime) were added into bacterial suspension at 37°C for 10 min in the dark. Bright-field microscopy images were acquired using a microscope with 100× oil immersion lens. Cell length and cell wall thickness quantitative measurements were made using Digimizer software (MedCalc, Ltd., Ostend, Belgium) (57). An unpaired *t* test was used for statistical analysis. The branched cells were not included in the cell length measurement analysis.

### Expression and purification of EnvZ_del_, MtrA, MtrA_D56E_, and MtrA_D56N_.

For EnvZ_del_, MtrA, MtrA_D56E_, and MtrA_D56N_ protein overproduction, E. coli BL21(DE3) cells transformed with pET-28a vector derivatives expressing EnvZ_del_, MtrA, MtrA_D56E_, and MtrA_D56N_ were grown overnight in Luria-Bertani (LB) medium with kanamycin at 37°C with shaking at 150 rpm. Cultures were diluted 1:100 and grown in fresh medium until the OD_600_ reached to 0.4 to 0.6. Next, 1 mM IPTG (isopropyl-β-d-thiogalactopyranoside) was added to the cultures for 16 h at 20°C to induce protein production. Cells were harvested by centrifugation at 8,000 × *g* for 10 min at 4°C. After the pellet was resuspended in 20 mL of lysis buffer (50 mM NaH_2_PO_4_, 300 mM NaCl, 10 mM imidazole, 5% glycerin [pH 8.0]), the cells were disrupted via sonication, followed by centrifugation at 12,000 × *g* for 30 min at 4°C. The purification of EnvZ_del_, MtrA, MtrA_D56E_, and MtrA_D56N_ proteins used a gravity-flow column prepacked with 1 mL of nickel-nitrilotriacetate (Ni-NTA) agarose (Qiagen). After being washed with 10 mL of wash buffer (50 mM NaH_2_PO_4_, 300 mM NaCl, 20 mM imidazole, 5% glycerin [pH 8.0]), the proteins were eluted by applying 500 μL of elution buffer three times (50 mM NaH_2_PO_4_, 300 mM NaCl, 250 mM imidazole, 5% glycerin [pH 8.0]). The proteins were then ultrafiltered using ultrafiltration buffer (50 mM Tris-HCl [pH 7.5], 300 mM NaCl, 1 mM dithiothreitol [DTT], 10% glycerol) and stored at −80°C for further experiments.

### Phosphorylation of MtrA by EnvZ_del_ kinase.

The MtrA was phosphorylated by EnvZ, as previously described (36). In brief, purified His-EnvZ_del_ was diluted with ultrafiltration buffer. The reaction premixture (total volume, 100 μL) for the autophosphorylation assay was composed of 50 mM Tris-HCl (pH 7.5), 50 mM KCl, 20 mM MgCl_2_, 1 mM DTT, and 30 μM diluted His-EnvZ_del_. The reaction was initiated by adding 10 μL of 100 mM ATP at a final concentration of 10 mM at room temperature. After a 1-h reaction, a phosphorylated form of His-EnvZ_del_ was obtained. The reaction mixture described above was then mixed with 120 μM diluted His-MtrA, followed by incubation at room temperature for 1 h. Lastly, the phosphorylated form of His-MtrA was prepared for EMSA.

### Electrophoretic mobility shift assay.

DNA fragment was amplified using the primers mraZDIGF and mraZDIGR containing DIG label (see Table S3). Then, a 10 pM concentration of purified DNA fragment was incubated with 0 to 5 μM MtrA, MtrA_D56E_, MtrA_D56N_, or MtrA-P in binding buffer [5 mM Tris-HCl (pH 7.6), 50 mM KCl, 0.5 mM EDTA, 5 mM (NH_4_)_2_SO_4_, 0.5 mM DTT, 0.1% Tween 20, 5 mM MgCl_2_, 5 mM CaCl_2_] at room temperature for 30 min. The reaction was stopped by adding 10× loading buffer (50 mM Tris-HCl [pH 6.8], 30% glycerol, 0.12% bromophenol blue). The reaction mixtures were electrophoresed on a 5% nondenaturing polyacrylamide gel for 3 h at 40 V. Next, the DNA and proteins were transferred to nylon membranes using semidry transfer method for 10 min at 10 V. The subsequent immunological detection was using a DIG High Prime DNA Labeling and Detection Starter Kit I (Roche, Mannheim, Germany) according to the manufacturer’s instructions.

### RNA isolation and sequencing.

The cultures were mixed immediately with 10% (vol/vol) STOP solution (90% [vol/vol] ethanol, 10% [vol/vol] phenol) for 10 min on the ice when grown to the midexponential phase. Next, the pH of the cultures was adjusted to pH 6 using HCl. The cultures were then pelleted by centrifugation at 8,000 × *g* for 20 min at 4°C. Subsequently, cells were ground in liquid nitrogen and resuspended with RNAiso Plus (TaKaRa), followed by the addition of chloroform. After centrifugation (12,000 × *g*, 15 min, 4°C), the upper layer of the phenol-chloroform was collected, and the same volume of isopropanol was added. The RNA was obtained by centrifugation (12,000 × *g*, 10 min, 4°C), followed by the addition of 75% ethanol to precipitate RNA again. The genomic DNA was removed with recombinant DNase I (TaKaRa) in combination with the recombinant RNase inhibitor (TaKaRa). The quality and integrity of total RNA was assessed with an Agilent 4200 bioanalyzer. Sequencing was performed using the Illumina NovaSeq6000 platform (Magigene, Ltd., Guangzhou, China). Three biological replicates per condition were used in RNA-seq experiments.

### Transcriptomic data analysis.

The differentially expressed genes (DEGs) were defined by a threshold of fold change ≥2 and a false discovery rate (FDR) of ≤0.05. Heatmaps and stacked columns were produced using Origin software. The Gene Ontology (GO) annotation of DEGs was obtained using Blast2GO software. Enrichment analysis of DEGs in GO pathways (*Q* ≤ 0.05) was performed using OmicShare tools.

### Data availability.

The transcriptomic raw sequence has been deposited to National Microbiology Data Center under accession number NMDC10018150.
