# Supplementary material for: The Bacterial MtrAB Two-Component System Regulates the Cell Wall Homeostasis Responding to Environmental Alkaline Stress
Source: Microbiol Spectr. 2022 Sep 8;10(5):e02311-22. doi: 10.1128/spectrum.02311-22 (PMC9602371; doi:10.1128/spectrum.02311-22)
Supplement: Supplemental file 1 — Supplemental material. Download spectrum.02311-22-s0001.pdf, PDF file, 2.9 MB [file spectrum.02311-22-s0001.pdf]

Supplementary Information

The bacterial MtrAB two-component system regulates the cell wall homeostasis responding to environmental alkaline stress

Xiaoyu Qin[1], Kaiduan Zhang[1], Yuzhao Fan[2], Hui Fang[1], Yong Nie[1]*, Xiao-Lei Wu[1,3,4]*

[1] College of Engineering, Peking University, Beijing 100871, China

[2] School of Resources and Environmental Engineering, Hefei University of Technology, Hefei 230009, China

[3] Institute of Ocean Research, Peking University, Beijing 100871, China

[4] Institute of Ecology, Peking University, Beijing 100871, China

*Corresponding author: Research Scientist, College of Engineering, Peking University.

Tel: +86 10-62759047; Fax: +86 10-62759047; E-mail: nieyong@pku.edu.cn

*Corresponding author: Professor, College of Engineering, Peking University.

Tel: +86 10-62759047; Fax: +86 10-62759047; E-mail: xiaolei_wu@pku.edu.cn

Figure S1

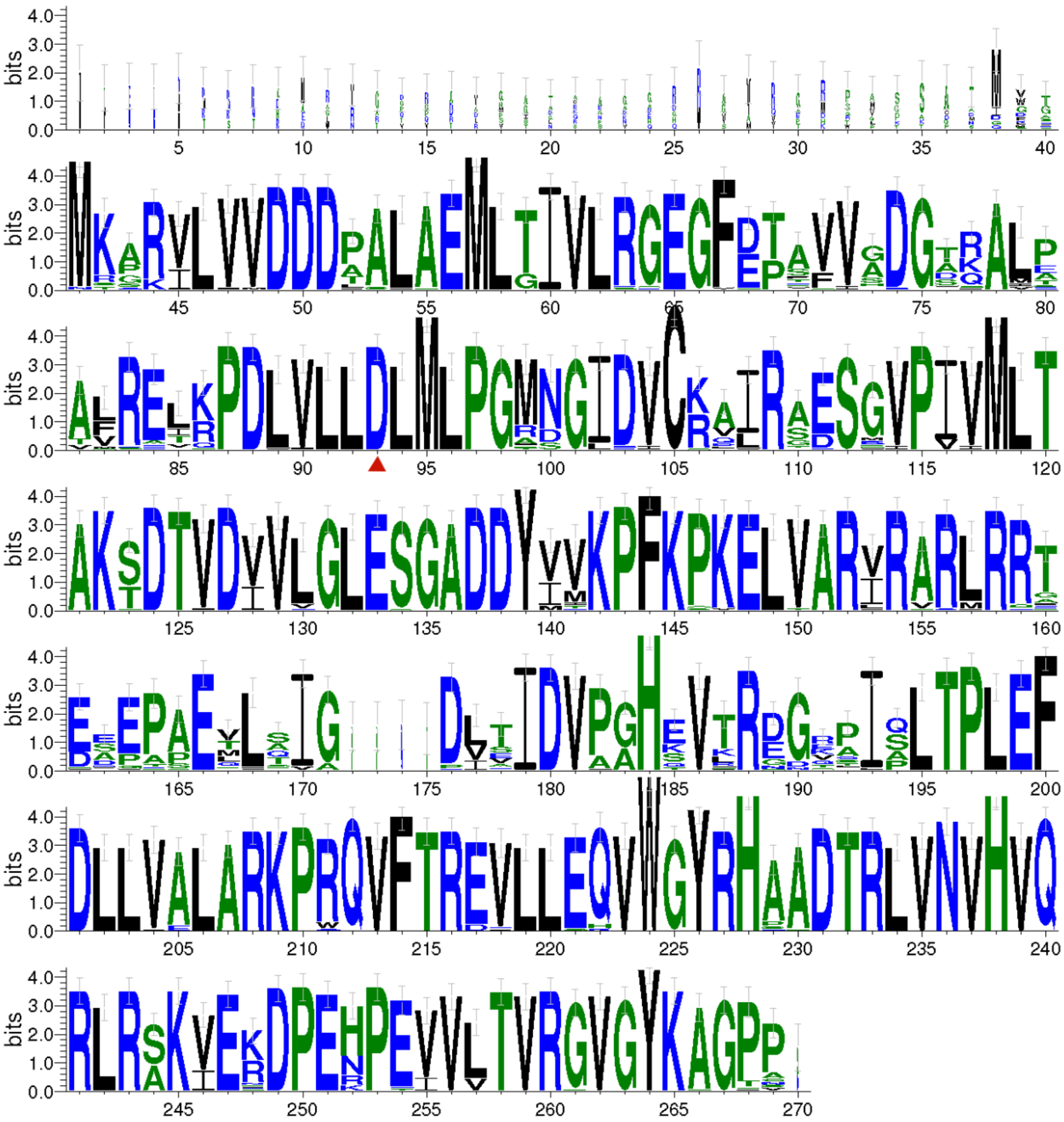

Fig S1. Comparison of the amino acid sequences of MtrA between *Dietzia* sp. DQ12-45-1b and other bacteria in Actinobacteria. The sequence logo was generated with WebLogo (http://weblogo.threeplusone.com/create.cgi). The red triangle represents the phosphorylation site.

Figure S2

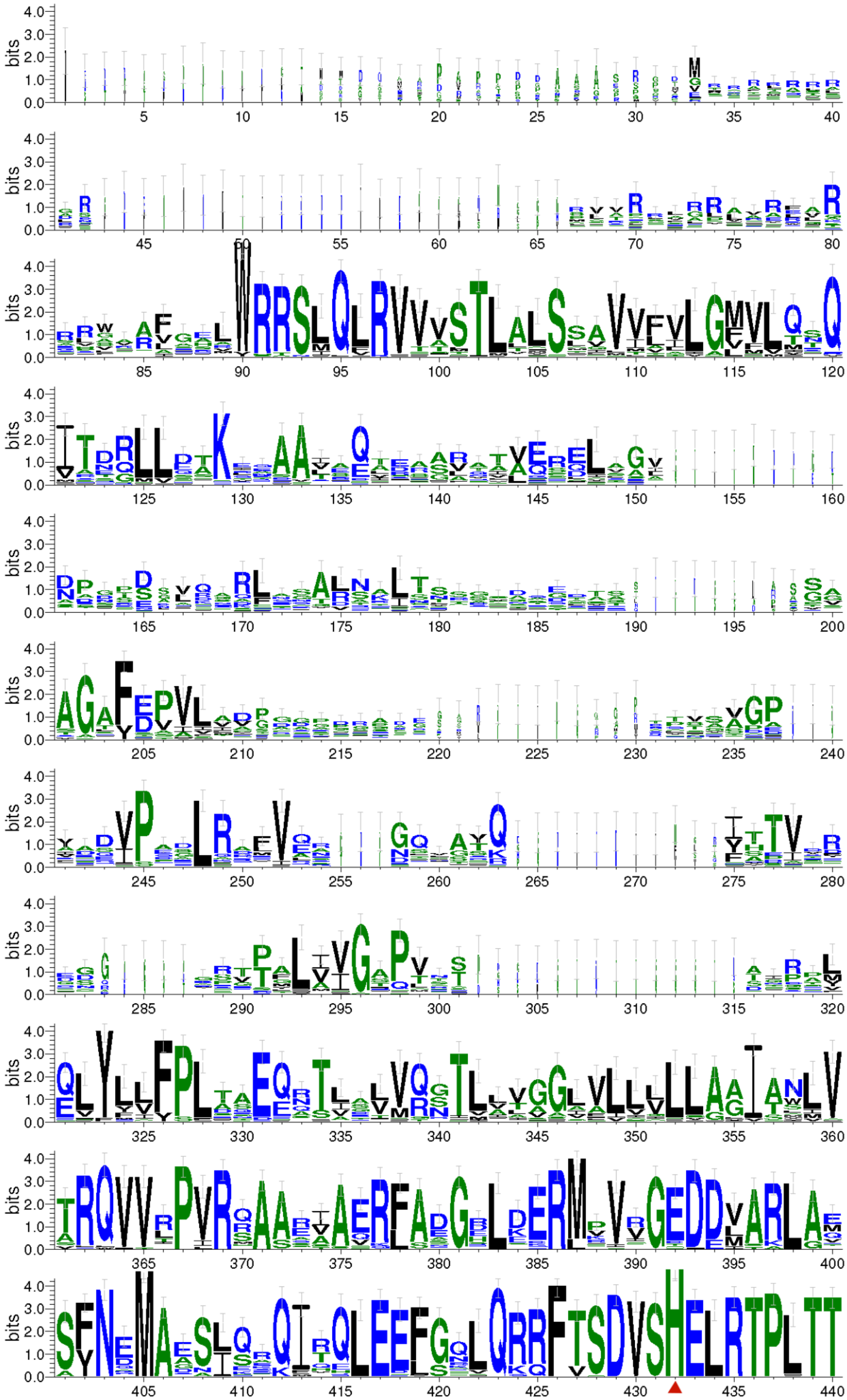

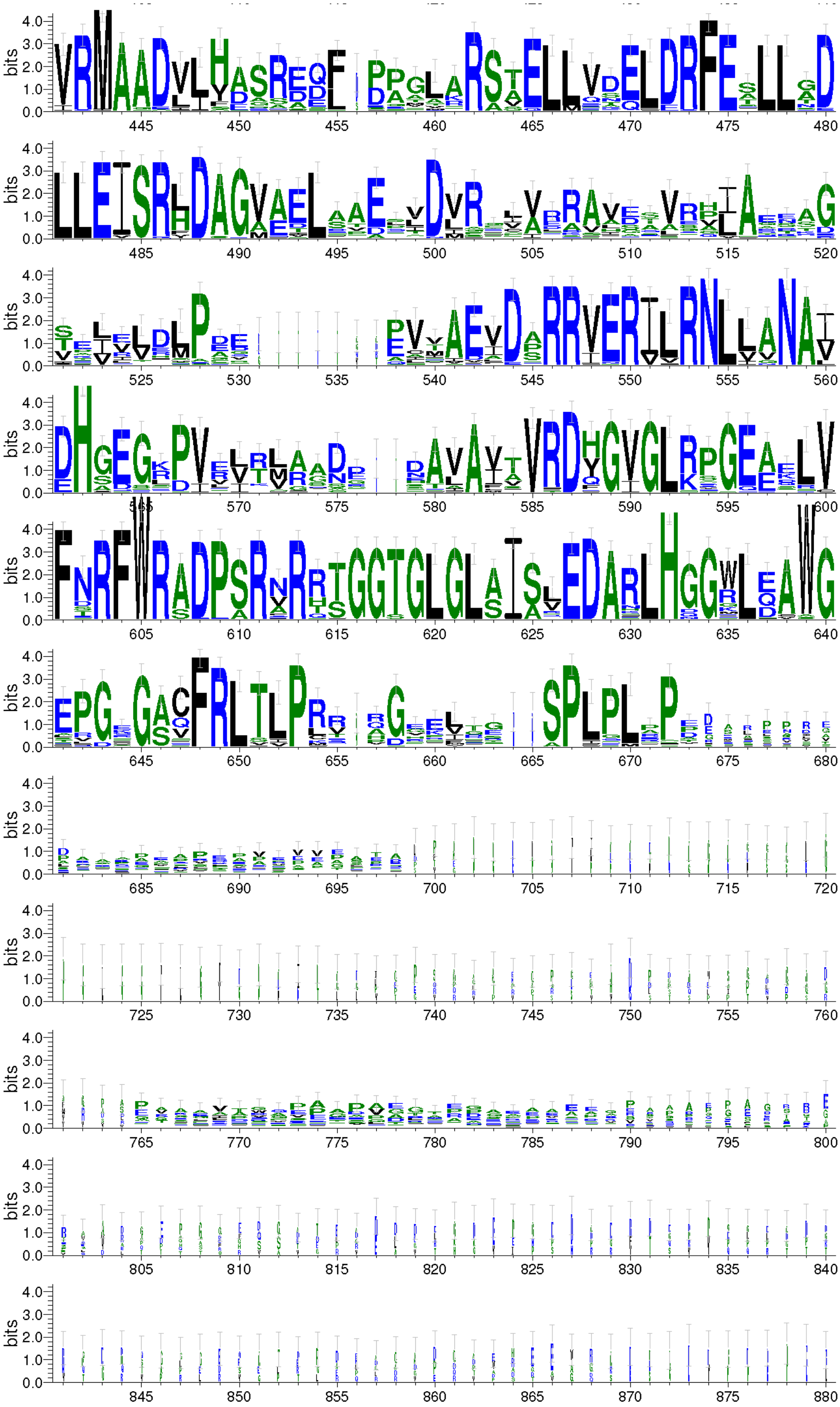

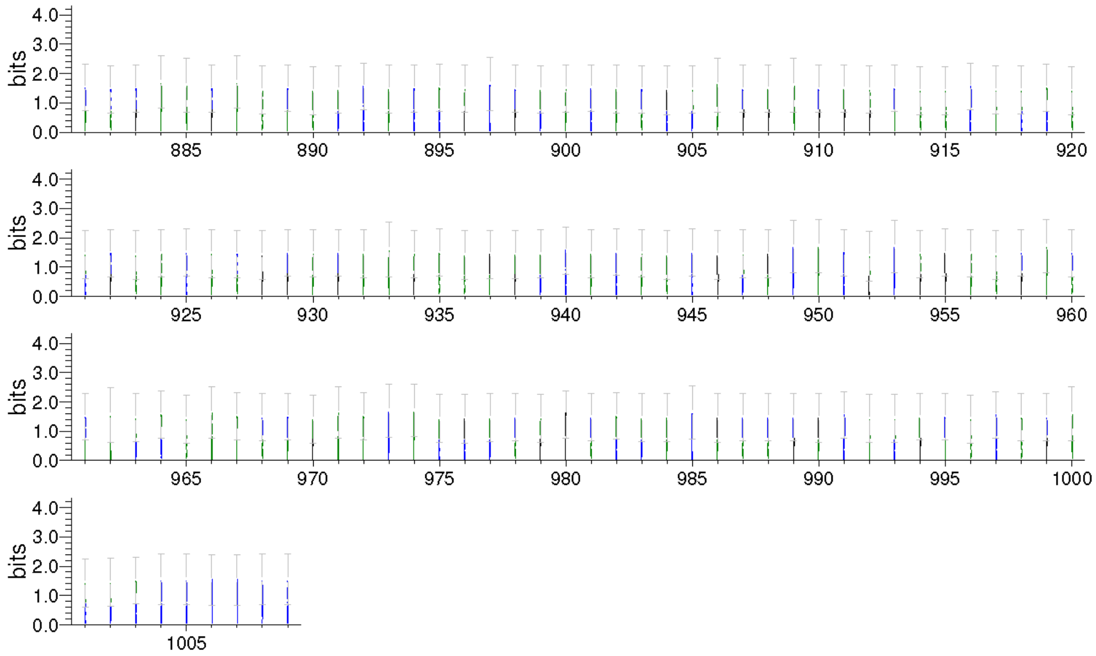

Fig S2. Comparison of the amino acid sequences of MtrB between *Dietzia* sp. DQ12-45-1b and other bacteria in Actinobacteria. The sequence logo was generated with WebLogo (http://weblogo.threeplusone.com/create.cgi). The red triangle represents the phosphorylation site.

Figure S3

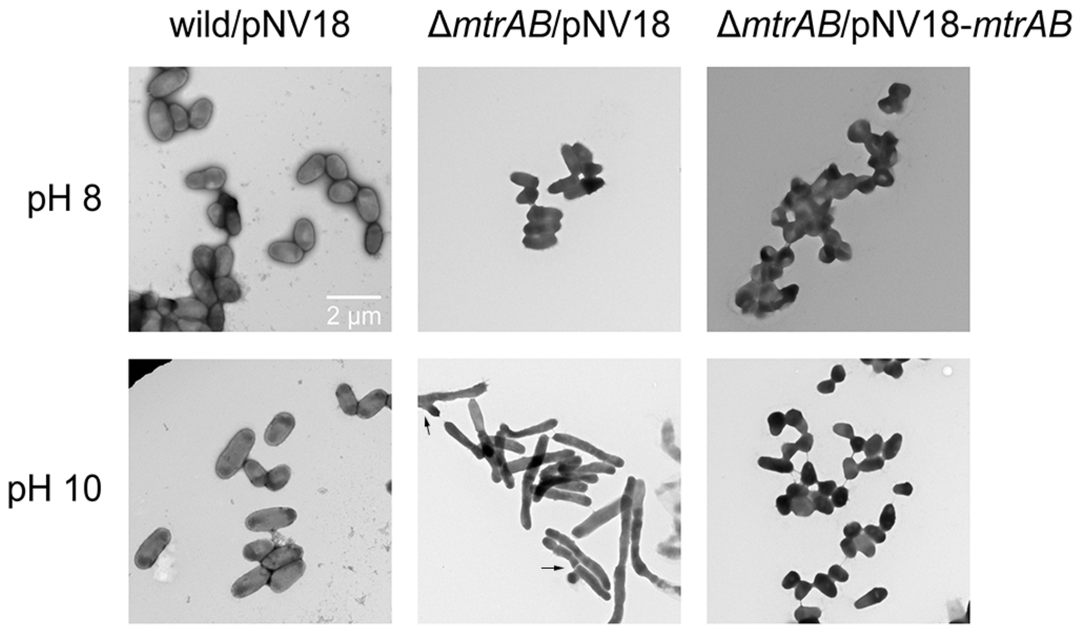

Fig S3. Cell morphology at different pH conditions. The representative micrographs of *Dietzia* sp. DQ12-45-1b wild/pNV18, Δ*mtrAB*/pNV18, and Δ*mtrAB*/pNV18-*mtrAB* by transmission electron microscope. Bars show the length in microns. Note the branched phenotype (marked with arrows) present in Δ*mtrAB*/pNV18 in pH 10.

Figure S4

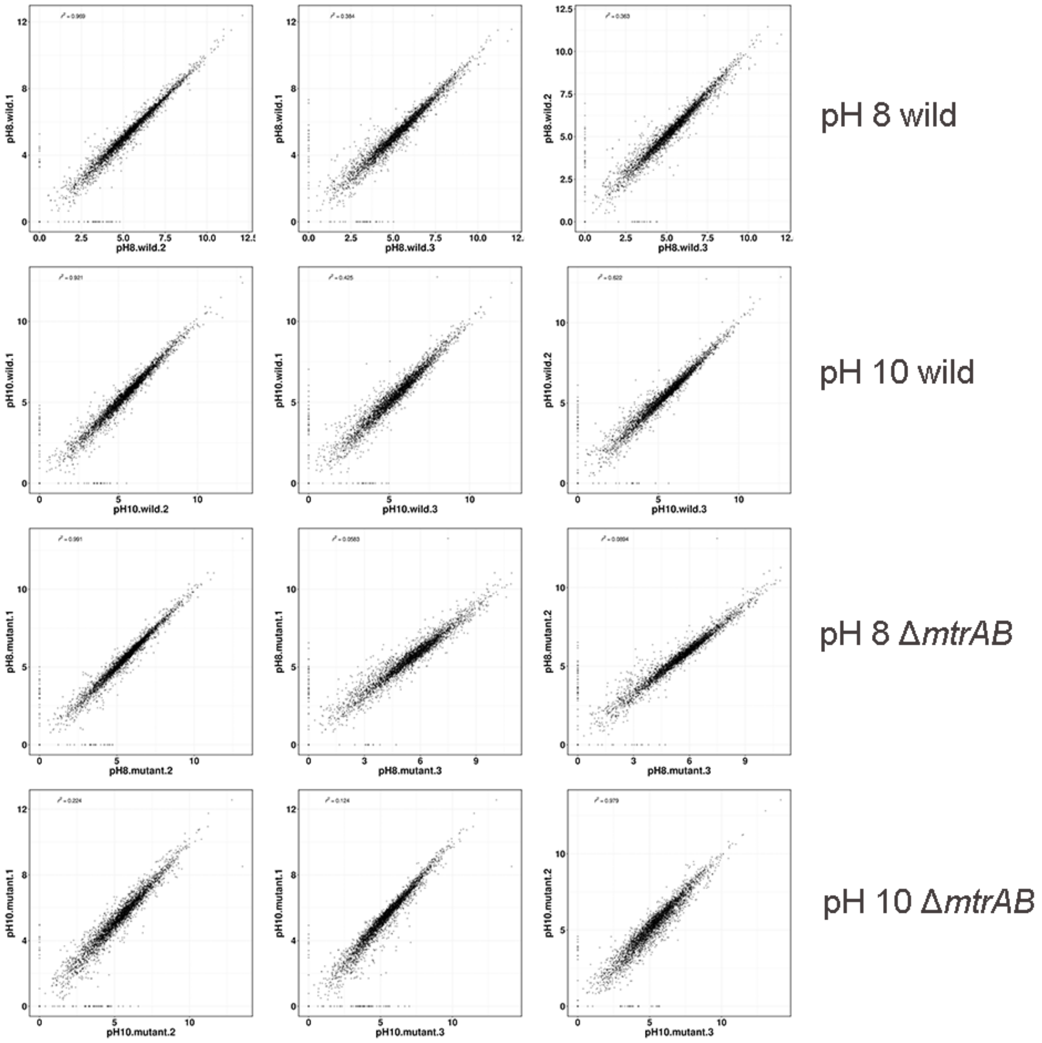

Fig S4. Scatter plots of consistency of transcriptomic data between parallel samples of *Dietzia* sp. DQ12-45-1b wild-type and Δ*mtrAB* mutant strains under the optimal and alkaline pH conditions.

Figure S5

A pH 10 wild/pH 8 wild

Up-regulated genes

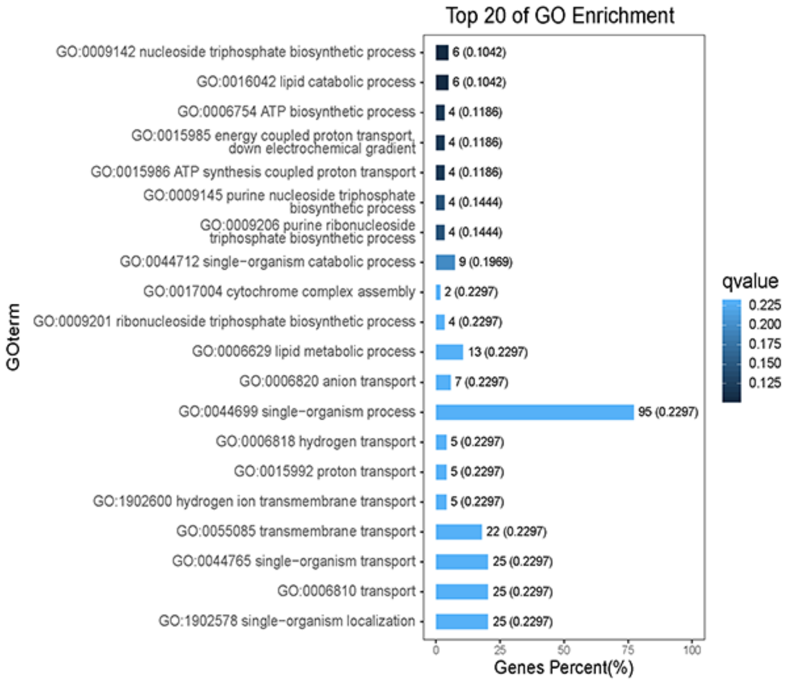

Down-regulated genes

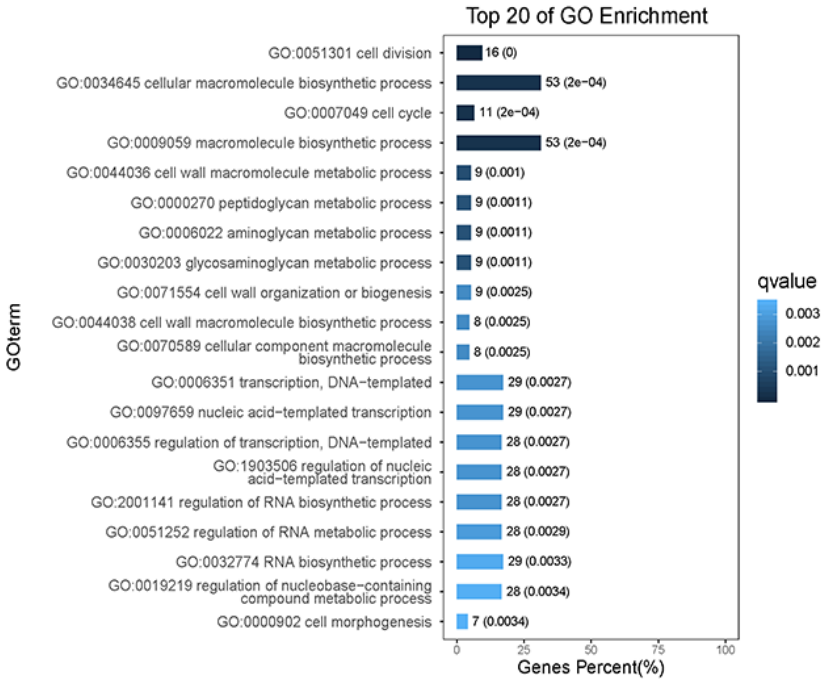

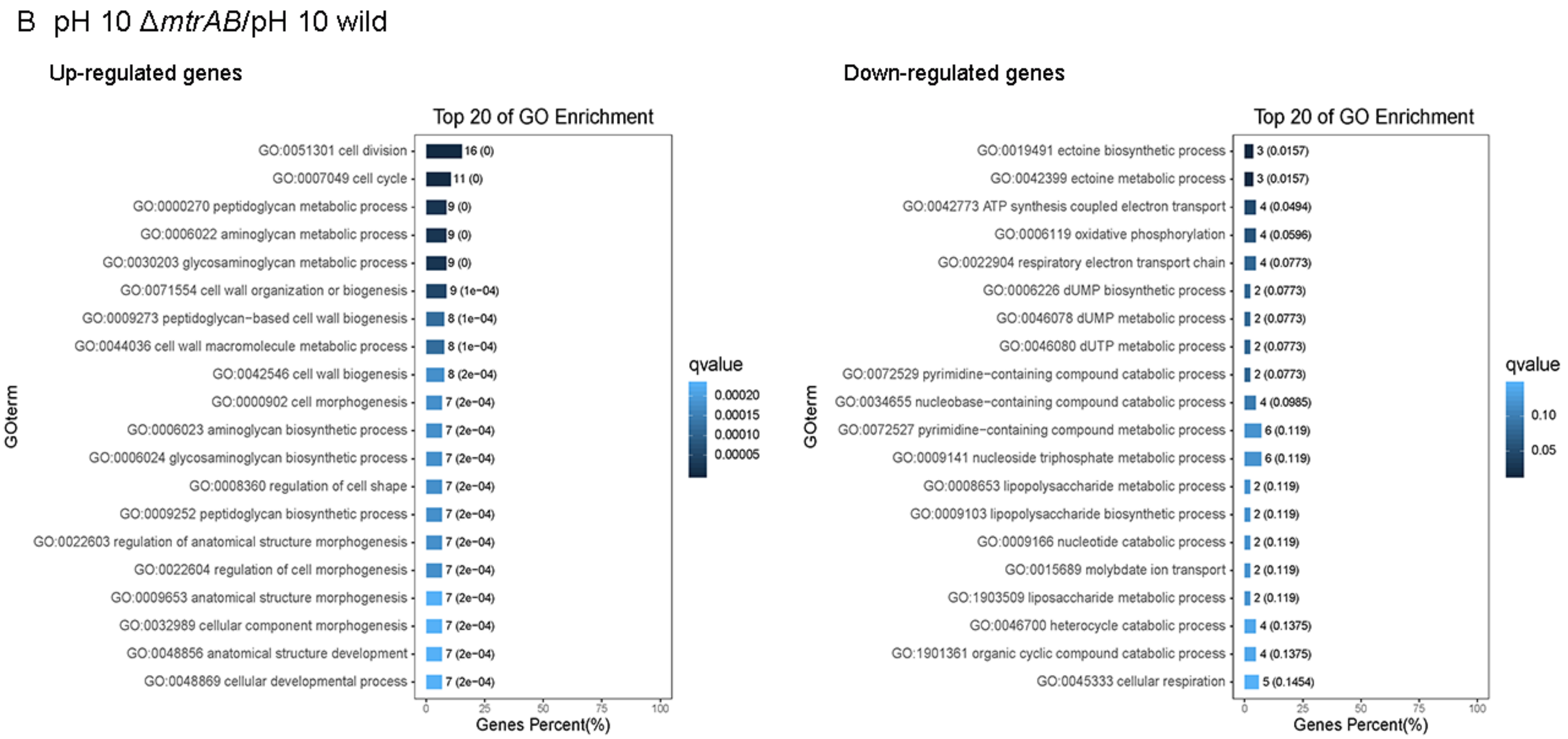

Fig S5. Gene Ontology (GO) classification and enrichment analysis. (A) Barplots of GO enriched from up-regulated genes and down-regulated genes of wild-type strain at pH 10 compared to pH 8 in Biological Process (BP) categories. (B) Barplots of GO enriched from up-regulated genes and down-regulated genes of Δ*mtrAB* mutant compared to wild-type strains at pH 10 in BP categories.

Figure S6

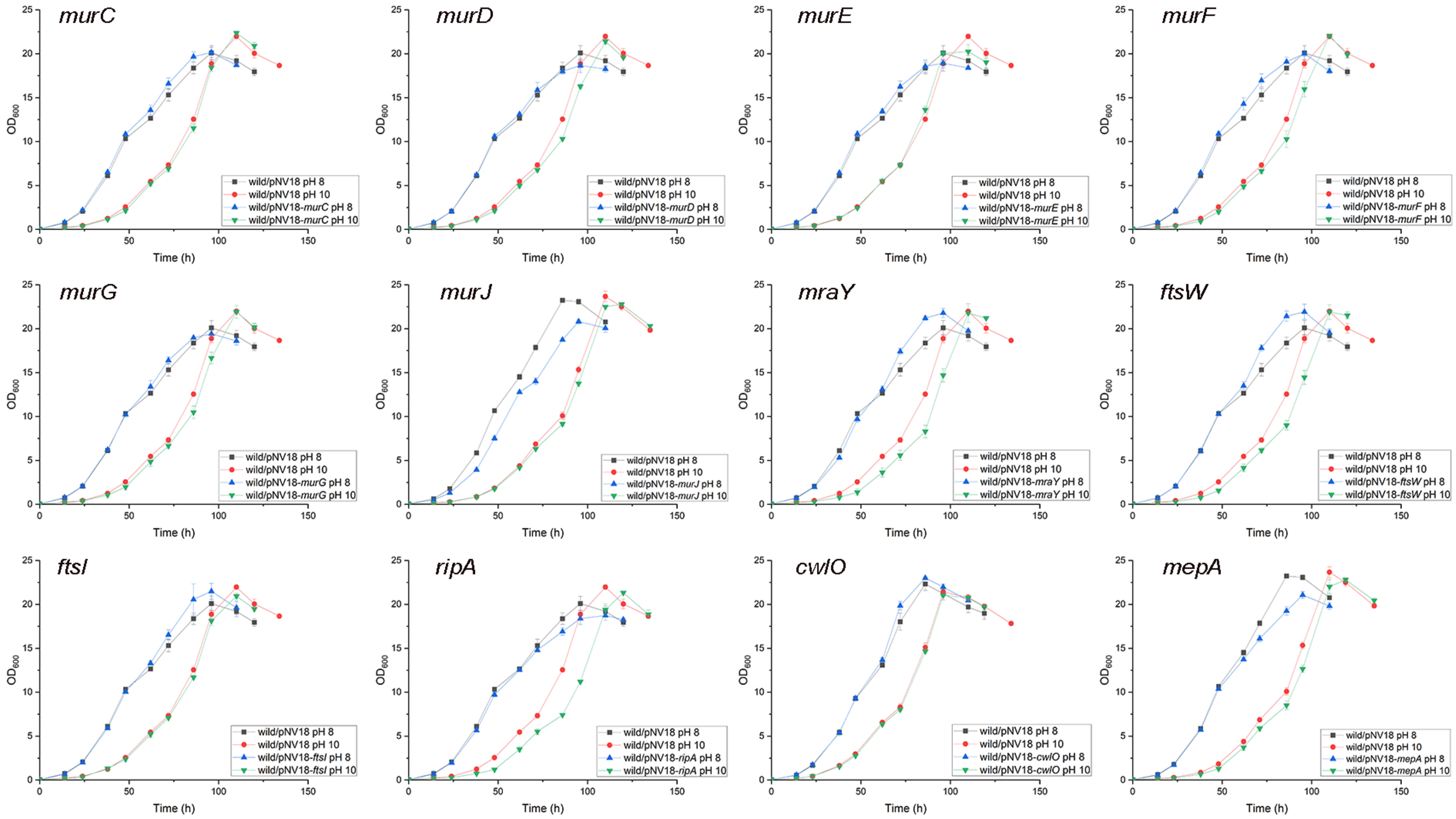
murC
murD
murE
murF
murG
murJ
mraY
ftsW
ftsI
ripA
cwlO
mepA
OD600
Time (h)
wild/pNV18 pH 8
wild/pNV18 pH 10
wild/pNV18-murC pH 8
wild/pNV18-murC pH 10
wild/pNV18-murD pH 8
wild/pNV18-murD pH 10
wild/pNV18-murE pH 8
wild/pNV18-murE pH 10
wild/pNV18-murF pH 8
wild/pNV18-murF pH 10
wild/pNV18-murG pH 8
wild/pNV18-murG pH 10
wild/pNV18-murJ pH 8
wild/pNV18-murJ pH 10
wild/pNV18-mraY pH 8
wild/pNV18-mraY pH 10
wild/pNV18-ftsW pH 8
wild/pNV18-ftsW pH 10
wild/pNV18-ftsI pH 8
wild/pNV18-ftsI pH 10
wild/pNV18-ripA pH 8
wild/pNV18-ripA pH 10
wild/pNV18-cwlO pH 8
wild/pNV18-cwlO pH 10
wild/pNV18-mepA pH 8
wild/pNV18-mepA pH 10

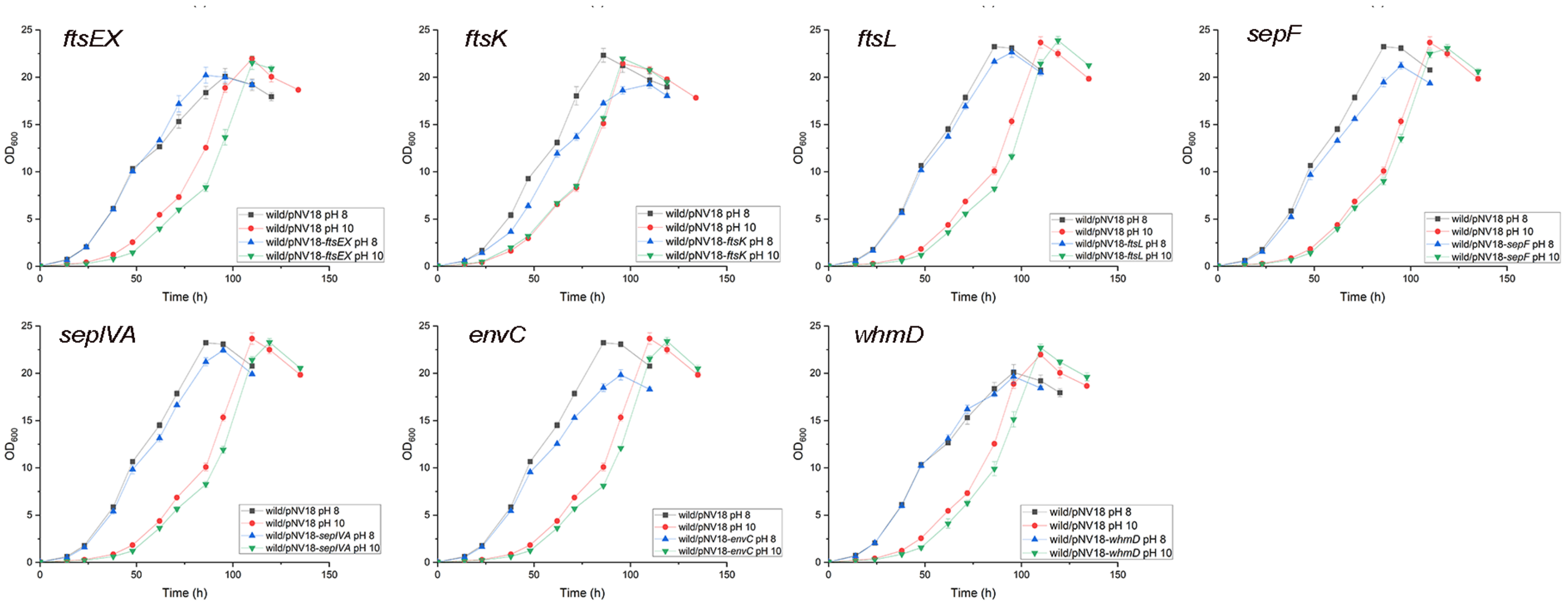

Fig S6. Growth curves of *Dietzia* sp. DQ12-45-1b wild-type strains overexpressing the genes involved in peptidoglycan homeostasis and cell division at different pH conditions. Black square and red circle represent wild-type strain carrying pNV18 at pH 8 and pH 10 respectively. Blue triangle and green inverted triangle mean wild/pNV18 with different genes at pH 8 and pH 10 respectively.

Figure S7

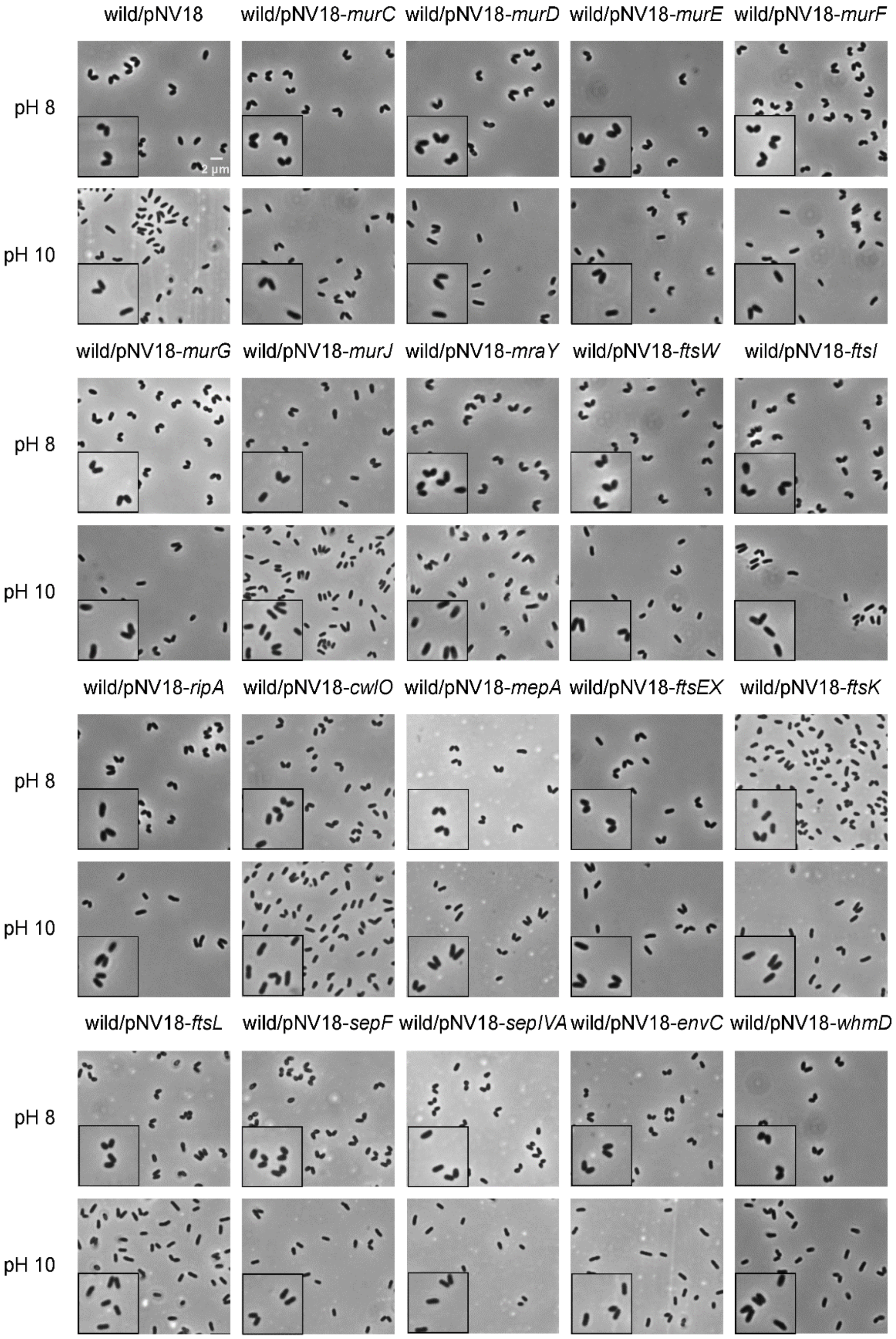

Fig S7. The representative micrographs of *Dietzia* sp. DQ12-45-1b wild-type strains overexpressing the genes involved in peptidoglycan homeostasis and cell division at different pH conditions.

Figure S8

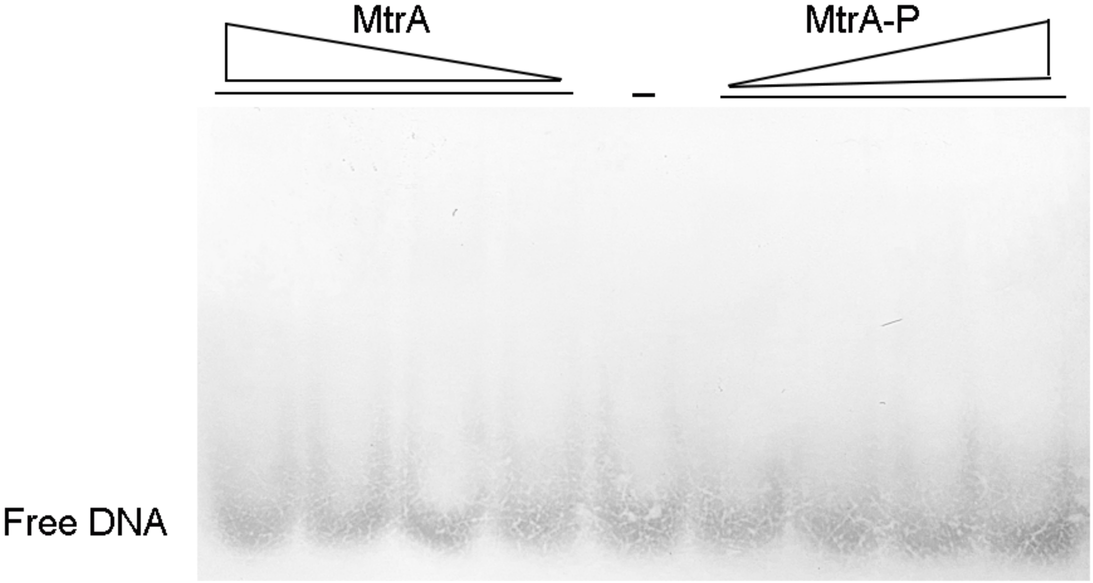

Fig S8. EMSA analysis of MtrA, MtrA-P binding to non-specific DNA (*Escherichia coli* DNA fragment) used as a negative control. MtrA, and MtrA~P at 0.5, 1, 2.5, and 5 μM concentration were incubated for 30 min with 0.1 μM of DIG-labeled DNA.

Table S1

Table S1 List of selected up- and down-regulated genes

Gene ID	Gene name	pH10 wild/pH8 wild		pH10 Δ*mtrAB*/pH10 wild		pH8 Δ*mtrAB*/pH8 wild		Gene product description
		Log_2FC	FDR	Log_2FC	FDR	Log_2FC	FDR	
Peptidoglycan synthesis								
GJR88_02065	*murE*	-1.45	0.000	2.44	0.000	0.21	0.326	UDP-N-acetylmuramoylalanyl-D-glutamate--2,6-diaminopimelate ligase
GJR88_02066	*murF*	-1.55	0.000	2.93	0.000	0.45	0.021	UDP-N-acetylmuramoyl-tripeptide--D-alanyl-D-alanine ligase
GJR88_02068	*mraY*	-1.34	0.000	2.85	0.000	0.52	0.003	phospho-N-acetylmuramoyl-pentapeptide-transferase
GJR88_02071	*murD*	-2.27	0.000	2.91	0.000	-0.46	0.028	UDP-N-acetylmuramoyl-L-alanyl-D-glutamate synthetase
GJR88_02073	*ftsW*	-2.70	0.000	2.64	0.000	-0.82	0.000	Cell division protein
GJR88_05430	*murJ*	-1.06	0.000	1.43	0.000	-0.10	0.676	Lipid II flippase
GJR88_02075	*murG*	-1.55	0.000	2.60	0.000	0.44	0.083	UDP-N-acetylglucosamine--N-acetylmuramyl-pyrophosphoryl-undecaprenol N-acetylglucosamine
GJR88_02077	*murC*	-1.53	0.000	2.65	0.000	0.56	0.003	UDP-N-acetylmuramate--L-alanine ligase

GJR88 _02062	*ftsI* (*pbpB*)	-1.58	0.000	2.61	0.000	-0.09	0.723	Penicillin-binding protein
Peptidoglycan hydrolysis								
GJR88 _02580	*ripA*	-2.39	0.000	1.85	0.000	1.63	0.000	Peptidoglycan endopeptidase
GJR88 _04713	NA	-2.59	0.000	3.89	0.000	1.06	0.000	1,4-beta-N-acetylmuramidase
GJR88 _02028	*cwlO*	-1.55	0.000	2.28	0.000	2.41	0.000	Peptidoglycan endopeptidase
GJR88 _00456	NA	-2.13	0.000	3.54	0.000	0.70	0.001	N-acetylmuramoyl-L-alanine amidase
GJR88 _03658	*mepA*	-0.41	0.125	1.84	0.000	2.09	0.000	Murein DD-endopeptidase
GJR88 _RS174 45	NA	-1.23	0.000	0.82	0.000	0.40	0.059	M23 peptidase domain-containing protein
GJR88 _03129	NA	-1.31	0.000	0.79	0.009	0.93	0.001	Membrane-bound lytic murein transglycosylase
Cell division								
GJR88 _02816	*ftsK*	-2.44	0.000	3.47	0.000	-0.43	0.032	Cell division protein
GJR88 _01576	*ftsE*	-1.55	0.000	1.24	0.000	-0.13	0.604	Cell division ATP-binding protein
GJR88 _01577	*ftsX*	-2.16	0.000	3.45	0.000	0.49	0.054	Cell division protein
GJR88	*ftsL*	-2.91	0.000	3.98	0.000	-0.32	0.137	Cell division protein

_02061								
GJR88 _02085	*sepF*	-1.41	0.000	1.18	0.000	0.34	0.108	Cell division protein
GJR88 _02952	*sepIVA*	-2.54	0.000	2.27	0.000	-0.81	0.000	Cell division protein
Related transcriptional regulators								
GJR88 _02058	*mraZ*	-3.33	0.000	4.68	0.000	-0.21	0.439	Transcriptional regulator
GJR88 _02225	*envC*	-1.36	0.000	2.90	0.000	2.61	0.000	Murein hydrolase activator
GJR88 _04446	NA	-1.64	0.000	2.06	0.000	1.04	0.000	LytR cell envelope-related transcriptional attenuator
GJR88 _01472	*whiB2* (*whmD*)	-2.03	0.000	2.75	0.000	0.77	0.001	WhiB family transcriptional regulator
GJR88 _03645	NA	-2.86	0.000	1.34	0.000	-0.93	0.002	Putative AsnC family transcriptional regulator
MtrAB TCS								
GJR88 _01482	*mtrA*	0.57	0.011	-12.85	0.000	-12.54	0.000	DNA-binding response regulator MtrA
GJR88 _01483	*mtrB*	0.76	0.000	-4.11	0.000	-4.30	0.000	Sensor histidine kinase MtrB
Monovalent cation/H^+ antiporter								
GJR88 _00766	*mrpG*	-1.21	0.000	-0.63	0.091	-1.83	0.000	Putative monovalent cation/H^+ antiporter subunit G
GJR88 _00768	*mrpF*	-1.23	0.021	0.52	0.480	-1.24	0.013	Putative monovalent cation/H^+ antiporter subunit F

GJR88 _00770	*mrpE*	-1.33	0.000	0.81	0.012	-1.10	0.000	Putative monovalent cation/H^+ antiporter subunit E
GJR88 _00771	*mrpD*	-1.19	0.000	0.30	0.184	-1.07	0.000	Putative monovalent cation/H^+ antiporter subunit D
GJR88 _00774	*mrpC*	-1.31	0.000	0.04	0.947	-1.34	0.000	Putative monovalent cation/H^+ antiporter subunit C
GJR88 _00777	*mrpA*	0.42	0.039	-0.71	0.000	-0.16	0.456	Putative monovalent cation/H^+ antiporter subunit A
GJR88 _04437	*mrpA*	0.80	0.000	-0.35	0.140	0.28	0.200	Putative monovalent cation/H^+ antiporter subunit A
GJR88 _04438	*mrpC*	-0.59	0.064	0.41	0.277	-0.84	0.005	Putative monovalent cation/H^+ antiporter subunit C
GJR88 _04440	*mrpD*	-0.54	0.023	0.67	0.006	-0.77	0.001	Putative monovalent cation/H^+ antiporter subunit D
GJR88 _04442	*mrpE*	-0.45	0.100	0.44	0.142	-1.14	0.000	Putative monovalent cation/H^+ antiporter subunit E
GJR88 _04443	*mrpF*	-1.31	0.001	0.31	0.571	-1.97	0.000	Putative monovalent cation/H^+ antiporter subunit F
GJR88 _04444	*mrpG*	-0.01	0.990	0.00	1.000	-0.69	0.005	Putative monovalent cation/H^+ antiporter subunit G

Table S2

Table S2 Strains and plasmids used in the study.

Strain or plasmid	Genotype and description	Source and reference
Dietzia strains		
Dietzia sp. DQ12-45-1b (CGMCC 1.10709)	Wild-type	(1)
DQ12-45-1b/pNV18	Wild-type harboring pNV18; Km^r	This study
DQ12-45-1bΔ*mtrAB*	DQ12-45-1b *mtrAB* deletion mutant; Sm^r	This study
DQ12-45-1bΔ*mtrAB/*pNV18	DQ12-45-1b *mtrAB* deletion mutant harboring pNV18; Km^r and Sm^r	This study
DQ12-45-1bΔ*mtrAB*/pNV18-*mtrAB*	DQ12-45-1bΔ*mtrAB* harboring pNV18-*mtrAB*; Km^r and Sm^r	This study
DQ12-45-1bΔ*mtrAB*/pNV18-*mtrA*	DQ12-45-1bΔ*mtrAB* harboring pNV18-*mtrA*; Km^r and Sm^r	This study
DQ12-45-1bΔ*mtrAB*/pNV18-$mtrA_{D56E}$	DQ12-45-1bΔ*mtrAB* harboring pNV18-$mtrA_{D56E}$; Km^r and Sm^r	This study
DQ12-45-1bΔ*mtrAB*/pNV18-$mtrA_{D56N}$	DQ12-45-1bΔ*mtrAB* harboring pNV18-$mtrA_{D56N}$; Km^r and Sm^r	This study
DQ12-45-1b/pJV	Wild-type harboring pJV; Km^r	This study
DQ12-45-1b/pJV-*mraZ*	Wild-type harboring pJV-*mraZ*; Km^r	This study

DQ12-45-1bΔ*mtrAB*/pJV	DQ12-45-1bΔ*mtrAB* harboring pJV; Km^r and Sm^r	This study
DQ12-45-1bΔ*mtrAB*/pJV-*mraZ*-AS	DQ12-45-1bΔ*mtrAB* harboring pJV-*mraZ*-AS; Km^r and Sm^r	This study
DQ12-45-1b/pNV18-*murC*	Wild-type harboring pNV18-*murC*; Km^r	This study
DQ12-45-1b/pNV18-*murD*	Wild-type harboring pNV18-*murD*; Km^r	This study
DQ12-45-1b/pNV18-*murE*	Wild-type harboring pNV18-*murE*; Km^r	This study
DQ12-45-1b/pNV18-*murF*	Wild-type harboring pNV18-*murF*; Km^r	This study
DQ12-45-1b/pNV18-*murG*	Wild-type harboring pNV18-*murG*; Km^r	This study
DQ12-45-1b/pNV18-*murJ*	Wild-type harboring pNV18-*murJ*; Km^r	This study
DQ12-45-1b/pNV18-*mraY*	Wild-type harboring pNV18-*mraY*; Km^r	This study
DQ12-45-1b/pNV18-*ftsW*	Wild-type harboring pNV18-*ftsW*; Km^r	This study
DQ12-45-1b/pNV18-*ftsI*	Wild-type harboring pNV18-*ftsI*; Km^r	This study
DQ12-45-1b/pNV18-*ripA*	Wild-type harboring pNV18-*ripA*; Km^r	This study
DQ12-45-1b/pNV18-*cwlO*	Wild-type harboring pNV18-*cwlO*; Km^r	This study
DQ12-45-1b/pNV18-*mepA*	Wild-type harboring pNV18-*mepA*; Km^r	This study
DQ12-45-1b/pNV18-*ftsEX*	Wild-type harboring pNV18-*ftsEX*; Km^r	This study
DQ12-45-1b/pNV18-*ftsK*	Wild-type harboring pNV18-*ftsK*; Km^r	This study
DQ12-45-1b/pNV18-*ftsL*	Wild-type harboring pNV18-*ftsL*; Km^r	This study
DQ12-45-1b/pNV18-*sepF*	Wild-type harboring pNV18-*sepF*; Km^r	This study
DQ12-45-1b/pNV18-*sepIVA*	Wild-type harboring pNV18-*sepIVA*; Km^r	This study

DQ12-45-1b/pNV18-*envC*	Wild-type harboring pNV18-*envC*; Km^r	This study
DQ12-45-1b/pNV18-*whmD*	Wild-type harboring pNV18-*whmD*; Km^r	This study
Escherichia coli strains		
BL21 (DE3)/pET-28a-$envZ_{del}$	BL21 (DE3) harboring pET-28a-$envZ_{del}$; Km^r	This study
BL21 (DE3)/pET-28a-*mtrA*	BL21 (DE3) harboring pET-28a-*mtrA*; Km^r	This study
BL21 (DE3)/pET-28a-$mtrA_{D56E}$	BL21 (DE3) harboring pET-28a-$mtrA_{D56E}$; Km^r	This study
BL21 (DE3)/pET-28a-$mtrA_{D56N}$	BL21 (DE3) harboring pET-28a-$mtrA_{D56N}$; Km^r	This study
Plasmids		
pNV18-DsRed.T4	*Dietzia-E.coli* shuttle vector (p45 promoter); Km^r	(2)
pNV18	Cloned from pNV18-DsRed.T4 without DsRed; Km^r	This study
pNV18-*mtrAB*	pNV18 + *mtrA* and *mtrB* from DQ12-45-1b; Km^r	This study
pNV18-*mtrA*	pNV18 + *mtrA* from DQ12-45-1b; Km^r	This study
pNV18-$mtrA_{D56E}$	pNV18 + *mtrA*(phosphorylation-mimic) from DQ12-45-1b; Km^r	This study
pNV18-$mtrA_{D56N}$	pNV18 + *mtrA*(phosphorylation-defective) from DQ12-45-1b; Km^r	This study
pJV53	*Dietzia-E.coli* shuttle vector (acetamide-inducible promoter); Km^r	(3)
pJV	Cloned from pJV53 without Che9cp60 and Che9cp61; Km^r	This study

pJV-*mraZ*	pJV + *mraZ* from DQ12-45-1b; Km^r	This study
pJV-*mraZ*-AS	pJV + *mraZ* in antisense orientation from DQ12-45-1b; Km^r	This study
pNV18-*murC*	pNV18 + *murC* from DQ12-45-1b; Km^r	This study
pNV18-*murD*	pNV18 + *murD* from DQ12-45-1b; Km^r	This study
pNV18-*murE*	pNV18 + *murE* from DQ12-45-1b; Km^r	This study
pNV18-*murF*	pNV18 + *murF* from DQ12-45-1b; Km^r	This study
pNV18-*murG*	pNV18 + *murG* from DQ12-45-1b; Km^r	This study
pNV18-*murJ*	pNV18 + *murJ* from DQ12-45-1b; Km^r	This study
pNV18-*mraY*	pNV18 + *mraY* from DQ12-45-1b; Km^r	This study
pNV18-*ftsW*	pNV18 + *ftsW* from DQ12-45-1b; Km^r	This study
pNV18-*ftsI*	pNV18 + *ftsI* from DQ12-45-1b; Km^r	This study
pNV18-*ripA*	pNV18 + *ripA* from DQ12-45-1b; Km^r	This study
pNV18-*cwlO*	pNV18 + *cwlO* from DQ12-45-1b; Km^r	This study
pNV18-*mepA*	pNV18 + *mepA* from DQ12-45-1b; Km^r	This study
pNV18-*ftsEX*	pNV18 + *ftsEX* from DQ12-45-1b; Km^r	This study
pNV18-*ftsK*	pNV18 + *ftsK* from DQ12-45-1b; Km^r	This study
pNV18-*ftsL*	pNV18 + *ftsL* from DQ12-45-1b; Km^r	This study
pNV18-*sepF*	pNV18 + *sepF* from DQ12-45-1b; Km^r	This study
pNV18-*sepIVA*	pNV18 + *sepIVA* from DQ12-45-1b; Km^r	This study

pNV18-*envC*	pNV18 + *envC* from DQ12-45-1b; Km^r	This study
pNV18-*whmD*	pNV18 + *whmD* from DQ12-45-1b; Km^r	This study
pET-28a	Overexpression vector; Km^r	Novagen
pET-28a-*$envZ_{del}$*	pET-28a + truncated *envZ* from *E.coli*; Km^r	This study
pET-28a-*mtrA*	pET-28a + *mtrA* from DQ12-45-1b; Km^r	This study
pET-28a-*$mtrA_{D56E}$*	pET-28a + *$mtrA_{D56E}$* from pNV18-*$mtrA_{D56E}$*; Km^r	This study
pET-28a-*$mtrA_{D56N}$*	pET-28a + *$mtrA_{D56N}$* from pNV18-*$mtrA_{D56N}$*; Km^r	This study

Table S3

Table S3 Primers used in the study.

Primer	Sequence (5’ → 3’)	Application
mtrABLF	TGCTGTTCGCCCTGGACC	Δ*mtrAB* mutant strain
mtrABLR	CCTTCATCCGTTTCCACGGTTCGTCGTCGACGACGAGGA	
mtrABRF	AAGCGTGCATAATAAGCCCTCTCGACCACTCGGAGTCCAA	
mtrABRR	ACCGGACGGAGAGAAGGTGT	
SmF	ACCGTGGAAACGGATGAAGG	
SmR	AGGGCTTATTATGCACGCTT	
fusionF	GTCCTGCTCGACCGCTACGT	
fusionR	CAGCACGGTGGCGCAGATCG	
mtrAF	CCC**AAGCTT**CGACATACACGCCGACACAA	Complementary plasmids
mtrAR	CCC**AAGCTT**TCACGAGACCGGACCGGCCT	
mtrABR	CCC**AAGCTT**TCACGAACGGACCTCCTCGG	
pNVF	CCC**AAGCTT**GGCACTGGCCGTCG	
pNVR	CCC**AAGCTT**GTGCCCGCTGAACTTCTTC	
mraZF	TACGAGATCGGCGGCCGCATCTGATCCCAGGAGGTGGCGG	*mraZ* overexpression plasmid
mraZR	GTCGGAATTCGCCGGGGCGCTTAGAATTCGATCCCCAGGG	

pJVF	GCGCCCCGGCGAATTCCGACG	All acetamide-inducible plasmids
pJVR	ATGCGGCCGCCGATCTCGTAA	
mraZASF	TACGAGATCGGCGGCCGCATCCCCAGGGATGTGCCCTCCA	*mraZ* antisense expression vector
mraZASR	GTCGGAATTCGCCGGGGCGCGACCCCAACGACTCCGAGCG	
pNVF2	AAGCTTGTGCCCGCTGAACT	Overexpression plasmids carrying the genes involved in peptidoglycan homeostasis and cell division
pNVR2	GGCACTGGCCGTCGTTTTAC	
murcF	AGTTCAGCGGGCACAAGCTTGCCGCCGGAAAGGCACGGCG	
murcR	GTAAAACGACGGCCAGTGCCTCATGACCCCGTCCCGCTCCC	
murdF	TTCAGCGGGCACAAGCTTCGAGCACATCGGGGTGACGGAC	
murdR	GTAAAACGACGGCCAGTGCCTCACCTCCCGGCGACCCGGCC	
mureF	AGTTCAGCGGGCACAAGCTTACGGAGAACGAGAGGCGGGT	
mureR	GTAAAACGACGGCCAGTGCCTCATGAGGTGTTGTCCTCCGT	
murfF	AGTTCAGCGGGCACAAGCTTCGGACGGAGGACAACACCTC	
murfR	GTAAAACGACGGCCAGTGCCTCATTCGGACATCTCCTCACC	
murgF	AGTTCAGCGGGCACAAGCTTATCCACCGGGGACGGGAGCG	
murgR	GTAAAACGACGGCCAGTGCCTCAACGCCGTGCCTTTCCGGC	
murjF	AGTTCAGCGGGCACAAGCTTCGTCGTCGAGGAGGTCTACGG	
murjR	GTAAAACGACGGCCAGTGCCTCACCGGGTGAGTTGTACCTC	
mrayF	AGTTCAGCGGGCACAAGCTTCGTGGTGAGGAGATGTCCGA	

mrayR	GTAAAACGACGGCCAGTGCCTCACCCCGATGTGCTCGCGAG
ftswF	AGTTCAGCGGGCACAAGCTTGGGTCGCCGGGAGGTAGGGC
ftswR	GTAAAACGACGGCCAGTGCCTCATCGCTCCCGTCCCCGGTG
ftsiF	AGTTCAGCGGGCACAAGCTTGACCTGCGAAAGGCCCCTCG
ftsiR	GTAAAACGACGGCCAGTGCCTCACTGCGCCTGCAGGATCAG
ripaF	AGTTCAGCGGGCACAAGCTTCGGTTATCGGAAGGTGAAAC
ripaR	GTAAAACGACGGCCAGTGCCTCAGGTCAGCCGGACGACCAT
cwloF	AGTTCAGCGGGCACAAGCTTCCAGGAAGAAAGAGGAAATC
cwloR	GTAAAACGACGGCCAGTGCCTCAGTAGTAGCGGGTCGCACC
mepaF	AGTTCAGCGGGCACAAGCTTGCCGTTCTGGCCGTCGCCGC
mepaR	GTAAAACGACGGCCAGTGCCTCAGCTGCCGAAGGGGATGGA
ftsexF	AGTTCAGCGGGCACAAGCTTTCGTGTGATCAGTGCTTCCC
ftsexR	GTAAAACGACGGCCAGTGCCTCACTGCCGAACCGTCACCCT
ftskF	AGTTCAGCGGGCACAAGCTTTCTGCGGGGTGGATGGGCGG
ftskR	GTAAAACGACGGCCAGTGCCTCAGCCCACCGGCGCCTCGTC
ftslF	AGTTCAGCGGGCACAAGCTTCGTATCGACGGGAGAACCCC
ftslR	GTAAAACGACGGCCAGTGCCTCAGTAGAGCGGGCCGGGGAC
sepfF	AGTTCAGCGGGCACAAGCTTATCAGGAAGGTCCCCGGAAG
sepfR	GTAAAACGACGGCCAGTGCCTCAGCGACGGAACGCGCCGTT

sepivaF	AGTTCAGCGGGCACAAGCTTTGAAGGGACGAGTGTGTACC	
sepivaR	GTAAAACGACGGCCAGTGCCTCAGTAGTCCCCGTACCCGTC	
envcF	AGTTCAGCGGGCACAAGCTTACCGAACTCCGAGGTTTCCC	
envcR	GTAAAACGACGGCCAGTGCCTCAGACGGCGACACCCTGCGC	
whmdF	AGTTCAGCGGGCACAAGCTTCGTAACACGAGGGGATGACC	
whmdR	GTAAAACGACGGCCAGTGCCTCACATCACGGCGTCACGCTT	
mtrAD56NF	CCTGCTGAACCTCATGCTCC	MtrA phosphorylation-defective and -mimic vectors
mtrAD56EF	CCTGCTGGAGCTCATGCTCC	
mtrAD56NR	CGAGACCGGACCGGCCTTGTAC	
pNVD56NF	GTACAAGGCCGGTCCGGTCTCG	
pNVD56NR	GGAGCATGAGGTTCAGCAGG	
pNVD56ER	GGAGCATGAGCTCCAGCAGG	
envZF	AAATGGGTCGCGGATCCGAATTCTTAAGCCATCAGATGGCGCA	$EnvZ_{del}$ expression vector
envZR	GCTCGAGTGCGGCCGCAAGCTTTTACCCTTCTTTTGTCGTGCCC	
pETF	AAGCTTGCGGCCGCACTCGAGCA	
pETR	GAATTCGGATCCGCGACCCATTTGC	
mtrAF	GGGAATTC**CATATG**GTGACAATGACCCCGAAGAT	MtrA expression vector
mtrAR	CCC**AAGCTT**TCACGAGACCGGACCGGCCT	
pETF2	CCC**AAGCTT**GCGGCCGCACTCGAGCACCACC	
pETR2	GGGAATTC**CATATG**GCTGCCGCGCGGCACCAGGC	

mraZDIGF	AGGTGGGGAGAAGTGGGGCG (5'-Digoxigenin)	EMSA
mraZDIGR	CCGCCACCTCCTGGGATCAG	

References

1. Wang X-B, Chi C-Q, Nie Y, Tang Y-Q, Tan Y, Wu G, Wu X-L. 2011. Degradation of petroleum hydrocarbons (C6–C40) and crude oil by a novel *Dietzia* strain. Bioresource Technology 102:7755-7761.
2. Szvetnik A, Bihari Z, Szabo Z, Kelemen O, Kiss I. 2010. Genetic manipulation tools for *Dietzia* spp. Journal of Applied Microbiology 109:1845-1852.
3. Van Kessel JC, Hatfull GF. 2007. Recombineering in *Mycobacterium tuberculosis*. Nature Methods 4:147-152.
